# Reversing the immunosuppressive microenvironment with reduced redox level by microwave-chemo-immunostimulant Ce–Mn MOF for improved immunotherapy

**DOI:** 10.1186/s12951-022-01699-w

**Published:** 2022-12-03

**Authors:** Zhiheng Zeng, Changhui Fu, Xiaohan Sun, Meng Niu, Xiangling Ren, Longfei Tan, Qiong Wu, Zhongbing Huang, Xianwei Meng

**Affiliations:** 1 Laboratory of Controllable Preparation and Application of Nanomaterials, Technical Institute of Physics and Chemistry, Chinese Academy of Sciences, Beijing, 100190 China; 2 CAS Key Laboratory of Cryogenics, Technical Institute of Physics and Chemistry, Beijing, 100190 China; 3 College of Biomedical Engineering, Sichuan University, Chengdu, 610065 China; 4 Department of Interventional Radiology, The First Hospital of China Medical University, Shenyang, 110000 China

**Keywords:** Ce–Mn MOF, Tumor microenvironment, Tumor-associated macrophages, Microwave therapy, Immunogenic cell death, Redox homeostasis

## Abstract

**Backgrounds:**

Reversing the immunosuppressive tumor microenvironment (TME) in the tumor is widely deemed to be an effective strategy to improve immune therapy. In particular, the redox balance in TME needs to be well controlled due to its critical role in mediating the functions of various cells, including cancer cells and immune-suppressive cells.

**Results:**

Here, we propose an efficient strategy to reshape the redox homeostasis to reverse immunosuppressive TME. Specifically, we developed a microwave-chemo-immunostimulant CMMCP to promote the infiltration of the tumor-T cells by simultaneously reducing the reactive oxygen species (ROS) and glutathione (GSH) and improving the oxygen (O_2_) levels in TME. The CMMCP was designed by loading chemotherapy drugs cisplatin into the bimetallic Ce–Mn MOF nanoparticles coated with polydopamine. The Ce–Mn MOF nanoparticles can effectively improve the catalytic decomposition of ROS into O_2_ under microwave irradiation, resulting in overcoming hypoxia and limited ROS generation. Besides, the activity of intracellular GSH in TME was reduced by the redox reaction with Ce–Mn MOF nanoparticles. The reprogrammed TME not only boosts the immunogenic cell death (ICD) induced by cisplatin and microwave hyperthermia but also gives rise to the polarization of pro-tumor M2-type macrophages to the anti-tumor M1-type ones.

**Conclusion:**

Our in vivo experimental results demonstrate that the microwave-chemo-immunostimulant CMMCP significantly enhances the T cell infiltration and thus improves the antitumor effect. This study presents an easy, safe, and effective strategy for a whole-body antitumor effect after local treatment.

**Supplementary Information:**

The online version contains supplementary material available at 10.1186/s12951-022-01699-w.

## Introduction

Due to the attractive advantage of the non-surgical in-situ killing of cancer tissues, deep tissue penetration, and high thermal efficiency, microwave (MW) hyperthermia has gained considerable attention as a safe, effective, and reliable therapeutic schedule in numerous solid tumors [1–4]. However, the practical application of MW therapy in the clinic is still limited by the high rate of recurrence after the treatment. The combination with other therapies is widely pursued to solve the above dilemma of MW hyperthermia therapy [5]. Among the potential methods, immunotherapy is one of the most attractive strategies because it can stimulate the immune systems for accurately tracking and erasing tumor cells [6–8]. However, the traditional MW hyperthermia therapy alone cannot activate the desired immunotherapy [9]. This is probably because hyperthermia therapy can result in immunogenic cell death (ICD) in the tumors, accompanied by the release of damage-associated molecular patterns (DMAPs), including surface-expressed calreticulin (CRT), high mobility group B1 (HMGB1) to realize the T cell-mediated immune response. However, the low production of DAMPs cannot elicit a robust immune response. Another reason could be that the solid tumors usually show the “cold” tumor microenvironment (TME), where abundant immune suppressive cells like protumoral M2-type tumor-associated macrophages (TAMs) and regulatory T cells (Tregs) suppress the cytotoxicity of anti-tumor T cells [10].

Reversing the immunosuppressive TME to activate the ICD is widely deemed to be an effective strategy to improve immune therapy [6, 11]. Nevertheless, the activation of ICD and the reverse of TME are usually compromised by a high redox state in TME, including the high level of reactive oxygen species (ROS), glutathione (GSH), and the long-term hypoxic state [12]. For instance, the high mobility group protein B1 (HMGB1) released outside the cell can serve as a "find me" signal, resulting in an immunogenic effect on enhancing the T cell infiltration to reverse “cold” tumors. However, the activated HMGB1 signal could be highly oxidized by the unique oxidative features in the TME [13–15], resulting in the severely weakened and even abolished activity of the HMGB1 [16, 17]. Besides, the abundance of TAMs could be polarized into the immunosuppressive M2-type macrophages by the increased ROS [18]. Furthermore, the activity of the Tregs is highly dependent on the antioxidant activity of GSH. As mentioned above, reshaping redox homeostasis is an efficient strategy to enhance immunotherapy [19–21]. However, previous studies have indicated that merely increasing or decreasing the ROS concentration in TME show a limited effect on promoting immunotherapy. A well regulation of the redox balance in the tumor will be favorable for activating immune therapy to improve the efficiency of MW thermal therapy.

Due to the large specific surface area, porosity, good biodegradability and adjustable performance, the metal–organic framework (MOF) materials exhibit great potential application in antitumor therapy by MW hyperthermia. In particular, the bimetallic MOF has attracted increasing attention because of its ability to provide two kinds of metal functions and more active sites [22–25]. It has been widely reported that the Ce^4+^-species possessed the antioxidative capacities to scavenge ROS due to the reversible redox of Ce^3+^/Ce^4+^ [25–29]. Furthermore, the high oxidation state of Mn can effectively reduce the activity of intracellular GSH to regulate redox signaling [30, 31]. Therefore, the rational construction of Ce and Mn-based bimetallic MOF is expected to show multiple functions to reshape TME and activate the immune system via redox regulation for a novel application in the field of multimodal immunotherapy. However, there is still no report of reprogramming the redox homeostasis of TME by simultaneously scavenging ROS and reducing GSH.

In this work, we designed a multifunctional microwave-chemo-immunostimulant based on a biodegradable matrix of bimetallic Ce–Mn MOF to achieve the combination of the MW hyperthermia, chemotherapy, and immune therapy for increasing anti-tumor T cells. The microwave-chemo-immunostimulant, CMMCP, was fabricated by coating polydopamine (PDA) on the surface of the chemotherapeutic drug cisplatin (CDDP) that was loaded into the bimetallic Ce–Mn MOF nanoparticles. We demonstrated that the resultant CMMCP can reprogram the redox environment by simultaneously ameliorating the excessive ROS and GSH and enhancing the concentration of O_2_. Thus the TAMs are effectively repolarized and the ICD was boosted. To the best of our knowledge, this is the first report that the therapeutic effect of tumor treatment can be greatly enhanced by improving immune therapy through reprogramming the redox homeostasis of TME.

## Results and discussion

### Characterization of CMMCP

**Fig. 1:**
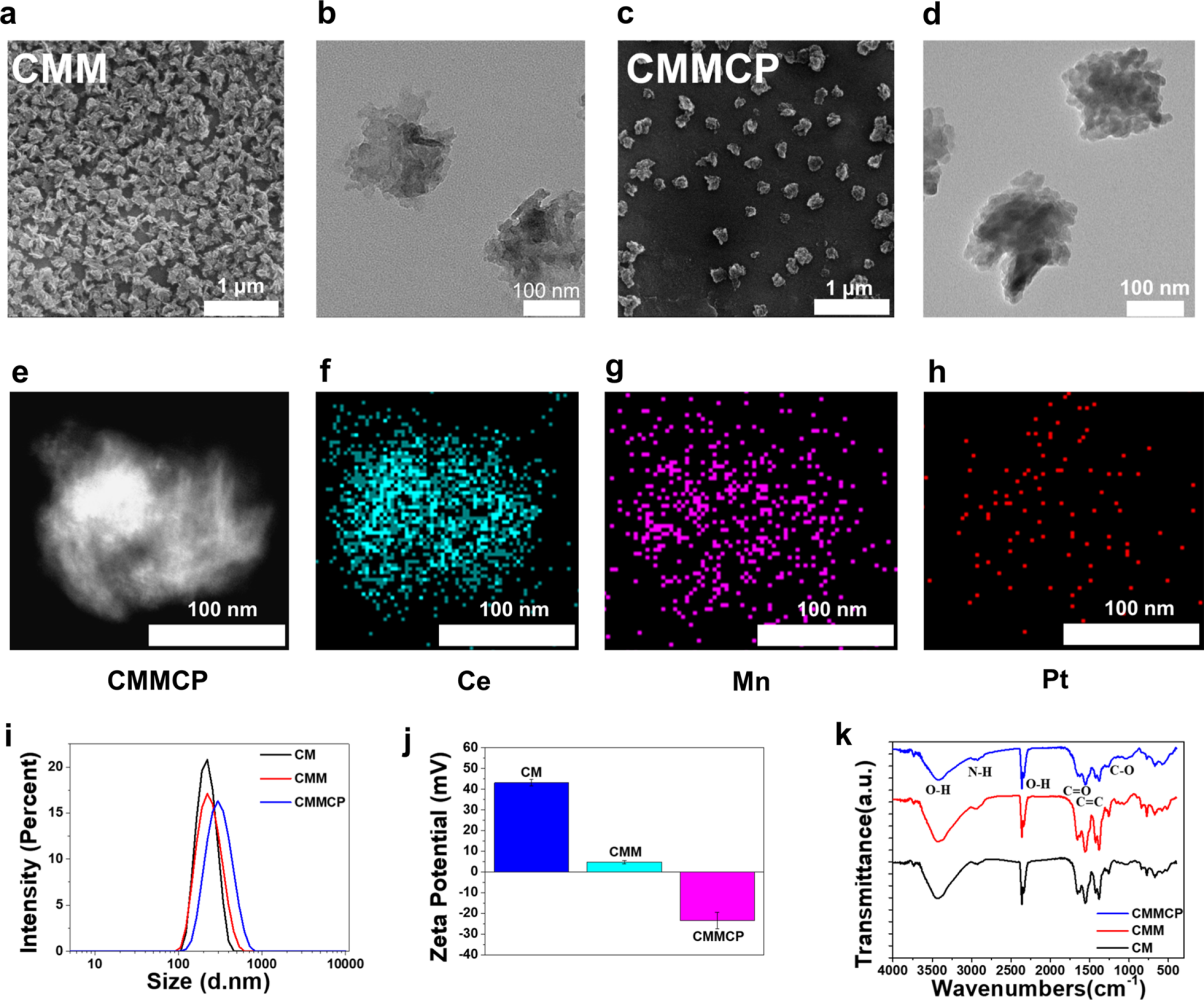
Characterization of CMM, and CMMCP. **a**, **b** SEM and TEM images for CMM. **c**, **d** SEM and TEM images for CMMCP. **e**–**h** Elemental mapping for CMMCP. **i**–**k** Size distribution, zeta potential, and FTIR spectra for CM, CMM, and CMMCP.

Specifically, CMMCP was prepared as follows. Firstly, Ce–Mn MOF nanoparticles (CMM) were synthesized by the one-step solvothermal method [25]. Then, CDDP was loaded into the CMM to obtain CMMC. Finally, PDA was coated on the surface of CMMC to increase the stability and control the release of CDDP [32–34]. Scanning electron microscope (SEM) and transmission electron microscope (TEM) observations revealed that CMM had the nanoflowers morphology with an average size of about 220 nm and with good dispersity and uniform particle size distribution, as shown in Fig. 1a, b. The Ce-MOF (CM) as referencing sample was synthesized in the same way without manganese ions. The CM showed a similar structure to CMM, as shown in Additional file 1: Fig. S1. We also observed that Mn-doping in CMM formed a more condensed shape compared with CM. The corresponding element mapping of the TEM image shows that CMMCP is composed of Ce, Mn and Pt, in which Pt was from CDDP. Mn was doped homogeneously in the main skeleton of CMM in Fig. 1e–h. XPS spectrum of CMMCP was performed. As shown in Additional file 1: Fig. S2a, b, the fitting of the high-resolution Pt 4f XPS spectrum also indicates the successful loading of cisplatin owing to the observation of two characteristic peaks at 72.6 eV (4f7/2) and 73.1 eV (4f5/2) of Pt^2+^ species. These values are very close to the 4f7/2 (72.8 eV) and 4f5/2 (76.1 eV) of the pristine cisplatin (Pt(NH_3_)_2_Cl_2_) [35, 36]. Moreover, the high-resolution XPS of Mn reveals the main peaks at 641.6 and 653.4 eV of Mn 2p are attributed to Mn 2p3/2 and Mn 2P1/2, respectively. The spin-energy separation of 11.8 eV is a typical value of Mn^3+^ in CMM (Additional file 1: Fig. S2c), indicating that the added Mn^2+^ was oxidized to a higher state during the preparation process [37].

We also characterized the particle size, zeta potential, and Fourier transforms infrared spectroscopy (FTIR) of CM, CMM, CMMC, and CMMCP, as shown in Fig. 1i–k. After doping Mn, the potential of CMM (+ 3 mV) was much lower than that of CM (+ 40 mV). The surface potential changed to a negative value (− 5 mV) after the loading of CDDP owing to the electrostatic interaction enabled by the localized uneven charge distribution and possible hydrogen bonding effect [38, 39]. After the coating of PDA, the potential of CMMCP was about − 23 mV. Dynamic light scattering measurement shows that the particle size of CMMCP had a slight increase as compared with that of CMM. The FTIR spectrum presented in Fig. 1k showed that the peak located at 1304 cm^−1^ and 2933 cm^−1^ were typically ascribed to the tensile vibration of the C–O bond and N–H between in 2-amino-terephthalic acid, the peak located at 3437 cm^−1^ was the tensile vibration absorption caused by hydrogen bonds between O–H molecules. In addition, the peak at 1624 cm.^−1^ was assigned to the vibration absorption peak of the aromatic ring from both the 2-amino-terephthalic acid ligand and PDA. Most of these peaks were mainly derived from the ligand constructed for the MOF, so they did not change significantly in the FTIR spectra of the CM, CMM, and CMMCP owing to the presence of the same skeleton. The strength of these peaks decreased slightly after coating PDA because of the partial shielding of infrared light by PDA modification [34].

### Degradation and drug release of CMMCP for reversing TME

**Fig. 2:**
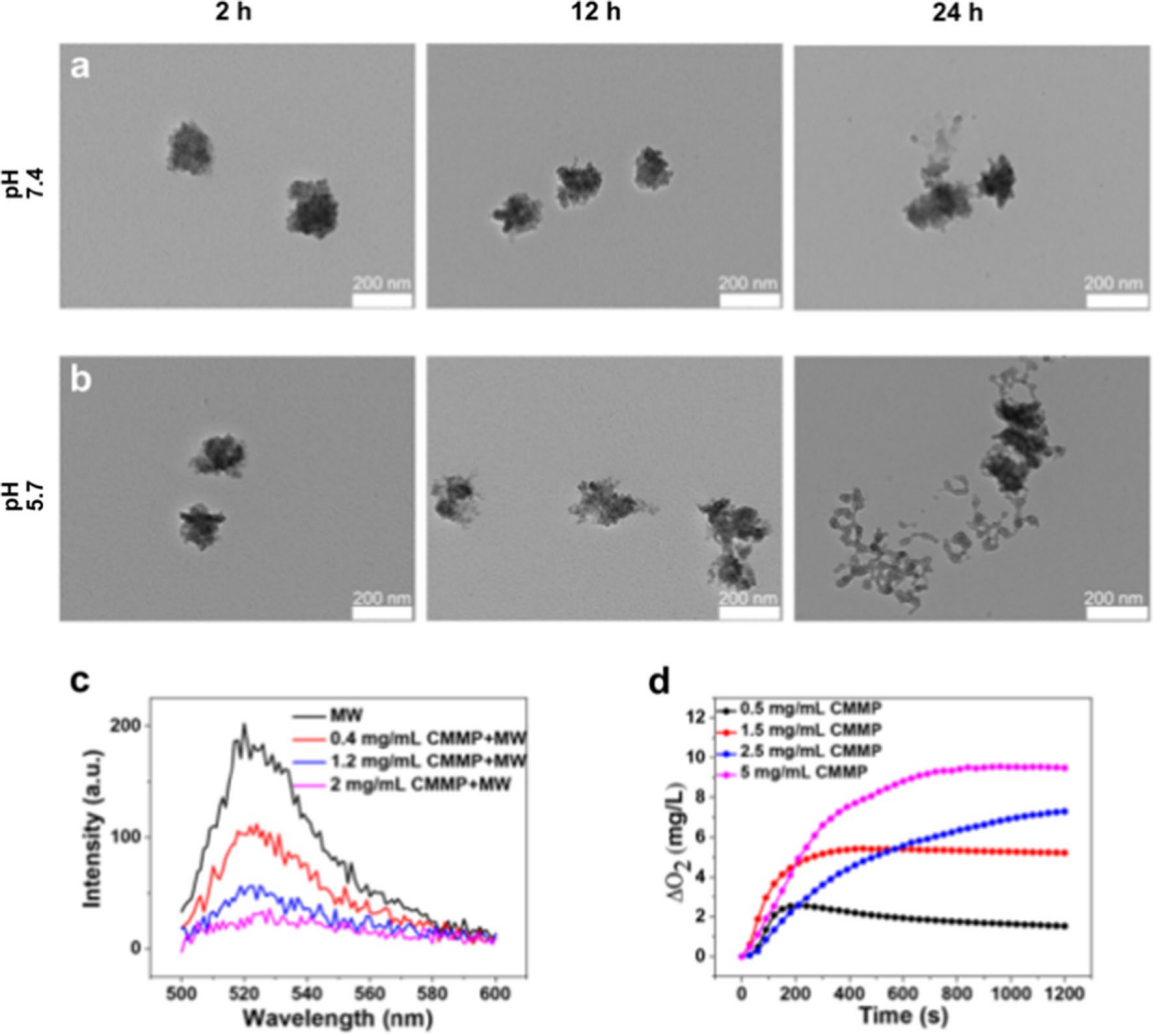
Degradation, ROS scavenging and O_2_ production of CMMP. **a**, **b** TEM images of degraded CMMCP after incubation in PBS with pH 7.4 and 5.7 for 2, 12 and 24 h. **c** ROS scavenging ability of CMMP at 0.4, 1.2, 2 mg/mL subjected to the MW irradiation at 0.9 W for 5 min. **d** O_2_ production of different concentrations of CMMP at 0.5, 1.5, 2.5 and 5 mg/mL after adding H_2_O_2_ solution

We tested the degradation of CMMCP under different pH values to evaluate the capability for drug release. The resultant CMMCP was dispersed in phosphate-buffered saline (PBS) with pH of 5.7 and 7.4 at 37 ℃. After incubating for 2, 6, 12 and 24 h, the morphology of precipitation was observed by TEM. As presented in Fig. 2a, b and Additional file 1: Fig. S1, CMMCP maintained the nanoparticle morphology with good dispersibility even after 24 h of incubation in neutral PBS solution (pH = 7.4). However, CMMCP exhibited slight degradation after 12 h of incubation in acidic PBS solution (pH = 5.7), and the degradation of CMMCP was more evident after a longer time. The different degradation behavior of CMMCP in the acidic and neutral environment may be attributed to the buffering effect mediated by the PDA layer. Such dependencies of degradation behavior on pH and incubation time indicated that the modification of PDA not only improves the stability of CMMC but also enables the capability for controllable drug release.

As mentioned above, the CMMCP exhibited good stability in a neutral physiological environment. Upon the CMMCP was delivered to the tumor region, the degradation of PDA occurs and leads to the release of CDDP owing to the acidic environment. We first evaluated the drug loading and release of CDDP from CMMCP in detail. CDDP was loaded on CMM, followed by encapsulation of a PDA shell. The encapsulated samples were used for testing the drug loading rate after shaking in DMF and the drug release rate within 24 h after being placed in acidic and neutral PBS. The corresponding curve of CDDP dispersed in DMF was calculated in Additional file 1: Fig. S3 according to the reported UV absorption method [40–43]. The drug loading rate was 12.3% calculated from the standard curve. With the same method, the CDPP was dispersed in acidic and neutral PBS to obtain the standard curves. Based on the standard curves of the drug in PBS, the calculated drug release rates of materials that shook in PBS with pH of 5.7 and pH of 7.4 at 37 °C for 24 h were 57.02% and 31.27%, respectively. This data further proves that the CMMCP is sensitive to acidic conditions and will be more conducive to the treatment in the TME.

The research showed that Ce^3+^ could convert hydroxyl radical (·OH) into O_2_, leading to the alleviation of the high concentration of ROS in TME. The ROS scavenging ability was tested using DCFH-DA as the ROS fluorescent probe. The results are shown in Fig. 2c and Additional file 1: Fig. S4. During the experiment, H_2_O_2_ was added. The strong fluorescence was observed for PBS with MW irradiation, indicating the decomposition process of H_2_O_2_ was accelerated after MW irradiation, thus the intensity of DCF was increased. Under the MW irradiation, the fluorescence intensities of CMM and CMMP were significantly lower than that of the control group with MW irradiation, the higher the concentration of material, the weaker the fluorescence intensity. Hence, the ROS could be decreased to a low level in the presence of CMM and CMMP, and the decrease of ROS was more evident with the assistance of MW irradiation.

It is well known that MW irradiation could improve redox kinetics. We assumed that the MW irradiation accelerated the redox reaction between Ce^3+^ and free radical as well as the Ce^3+^/Ce^4+^ redox reaction. It is thus expected that using the Ce-based MOF allowed to possibly simultaneously solve the issues of ·OH and hypoxia in TME. However, the removal of ·OH alone is not enough to reverse TME because the high concentration of H_2_O_2_ in the tumor can give rise to the production of ·OH and the abnormal oxidizing environment at the tumor site. Therefore, the removal of H_2_O_2_ is equally important. In this regard, we doped the Ce-based MOF with Mn^2+^ to convert H_2_O_2_ into ·OH through the Fenton-like reaction. The produced ·OH could also serve as a reactant to promote the redox of Ce^3+^.

As confirmed by XPS, the added Mn^2+^ was oxidized to a higher state during the preparation process. The higher oxidation state of Mn is in favor to reduce the GSH content in the tumor through a Redox reaction [31, 37] enabling the regulation of high reducibility in TME. The 5,5′-dithiobis (2-nitrobenzoic Acid) (DTNB) was further explored to detect the concentration of GSH in vitro. The DTNB can react with GSH to produce a yellow product with maximal absorption at 412 nm for the UV spectrum. As illustrated in Additional file 1: Fig. S3 and S5, the clearance rate of GSH gradually increased with the increase of CMMP concentration, indicating that CMMP can deplete GSH to reverse TME.

Several studies have demonstrated that the O_2_ generation properties of the material can relieve hypoxia in the tumor site. The potential capability of producing O_2_ by CMMCP was measured by dissolved oxygen testing. As shown in Fig. 2d and Additional file 1: Fig. S6, the O_2_ content produced by CM (10 mg) was only 3.75 mg/L, indicating that CM can promote the production of O_2_. In the case of CMM, the accumulative amount of O_2_ was increased to 12 mg/L, which is fourfold higher than that of CM owing to the improved generation of ROS owing to the Fenton-like reaction enabled by Mn^2+^. After coating PDA at the surface, the generated O_2_ of CMMP was decreased to 9 mg/L. The decreased O_2_ generation was attributed to the shielding effect of PDA. Overall, using the Ce–Mn MOF is expected to reverse the TME via conversing H_2_O_2_ and ·OH into O_2_ as well as decreasing the content of GSH, creating a favorable environment for ICD therapy and the repolarization of M2 macrophages to M1.

### Endocytosis and therapeutic effect in vitro

**Fig. 3:**
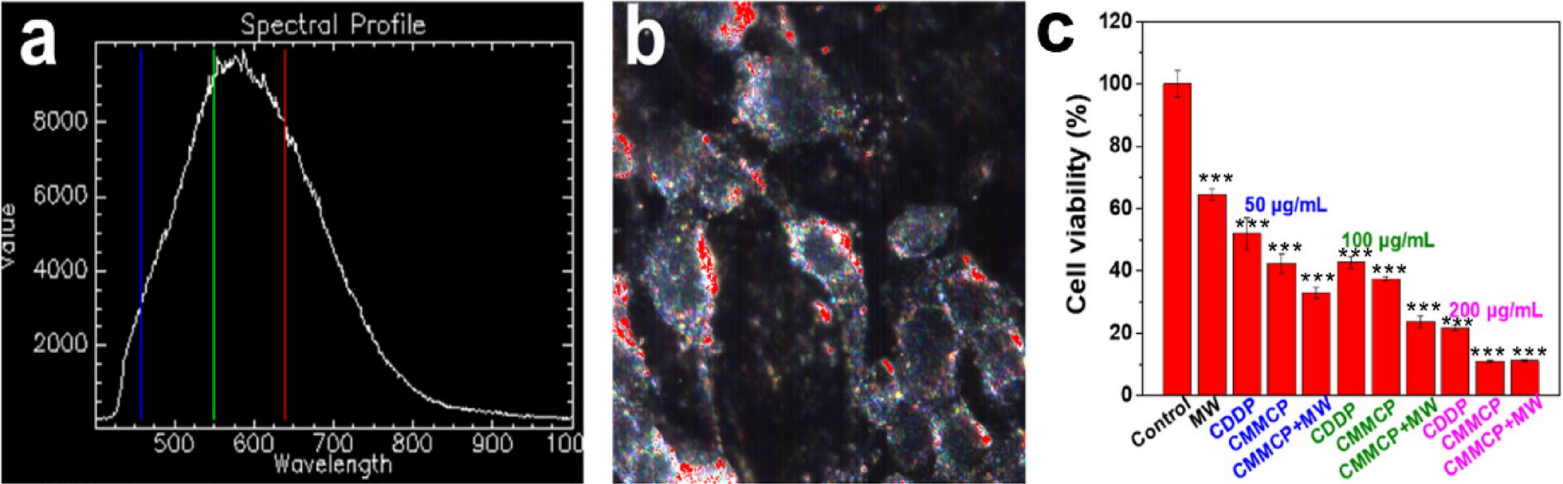
Hyperspectral observation of the treatment for 4T1 cells with CMMP. **a** Dark-field hyperspectral image of CMMP, the RGB vertical lines represented the "true color" wavelengths: red = 640 nm, green = 550 nm and blue = 460 nm. **b** Hyperspectral images for cells mapped with CMMP at 100 μg/mL. **c** Cell viability of 4T1 cells treated with MW, CDDP and CMMCP plus MW. Different concentrations of CMMCP (50, 100 and 200 μg/mL) and corresponding CDDP (6.15, 12.3, and 24.6 μg/mL) were incubated with 4T1 cells for 24 h and then cell viability was detected. *** indicates *p* < 0.001

We used hyperspectral imaging to study the endocytosic effect of CMMP. Hyperspectral imaging is increasingly developing as a well-established imaging technique based on total spectra acquisition and analysis. Unlike other imaging techniques, it can be used to observe and spectrally characterize nanoparticles without any fluorescent labeling or other sample preparation in vitro cell and ex vivo tissue. It allows for spectral mapping and confirmation of nanoparticle uptake in cells [44, 45]. Herein, the characteristic spectral peaks of CMMP in vitro were collected to merge with the same characteristic peaks in cells to label the location of CMMP. The characteristic hyperspectrum of CMMP shows the typical peaks at ~ 600 nm (Fig. 3a). As shown in Additional file 1: Fig. S7a and Fig. 3b, the cell morphology can be seen in the control cells and those treated with CMMP for 12 h. The black sphere and white region were identified as the nucleus of cells and the cytoplasm around the nucleus, respectively. The merged area was set as red to reveal the distribution of CMMP. In comparion to the control group, the red and white parts overlapped completely in cells treated CMMPs. This indicates that the CMMP nanoparticles can be endocytosed and are mostly distributed in the cytoplasm.

As we mentioned, due to the instability of CMM in the physiological environment and biotoxicity, CMM was coated with PDA to improve the stability and control the drug release. Here, the biocompatibility of CMM and CMMP in vitro was estimated by co-incubating with 4T1 and L929 cells for 24 h (Additional file 1: Fig. S7b, c). The results demonstrated that cell viability of CMM above 50 μg/mL was lower than 80% due to the rapid degradation of CMM. However, after the coating of PDA, cell activity remained above 85% even at the concentration of 200 μg/mL for 24 h of co-incubating, suggesting that PDA was favorable for improving the biocompatibility of CMM.

Afterward, we evaluated the treatment effect of CMMCP for tumors. Different concentrations of CMMCP (50, 100 and 200 μg/mL) and the corresponding CDDP (6.15, 12.3, and 24.6 μg/mL) were incubated with 4T1 cells for 24 h. As shown in Fig. 3c, the cell viability of the MW (0.9 W, 5 min) group reduced to about 65% compared with the control group. The 4T1 cells in the group with CMMP alone showed nearly unchanged activity due to the good biocompatibility of CMMP, but the cell viability for CMMCP at the 50 μg/mL group decreased to about 45%. The reason caused lower cell viability in CMMCP compared with CDDP group. The reason for the better therapeutic effect of CMMCP than CDDP can be that these components of ROS and GSH in the TME can decrease the reactivity of CDDP by reacting with Pt(II) [46, 47]. CMM could decrease ROS and GSH in the tumor and produced O_2_, which could improve the efficiency of CDDP as the tumor was more sensitive to CDDP under the reversed TME. With the MW irradiation, the cell activity in CMMCP group was further reduced to about 33% because the MW significantly promoted the elimination of ROS and also produced a moderate heating effect. Similar treatment on the cell viability was also observed for the group with CMMCP at 100 and 200 μg/mL. As the concentration of the CMMCP increased to 200 μg/mL, the viability of 4T1 cells decreased to only 10%, so there was no significant difference in CMMCP (200 μg/mL) before and after MW irradiation.

### ROS scavenging, O_2_ production, and GSH depletion by CMMP in vitro

**Fig. 4:**
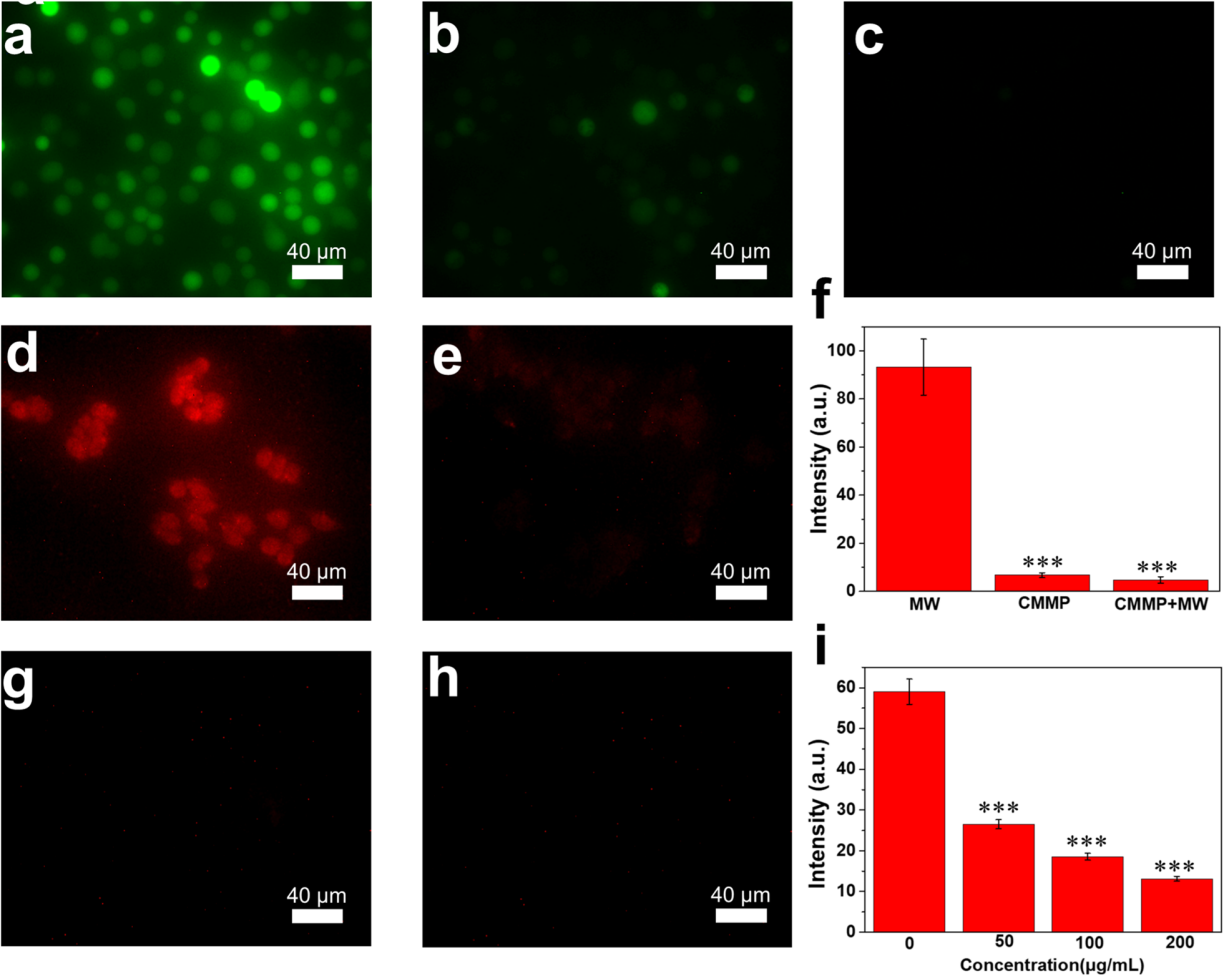
ROS scavenging and O_2_ production in vitro. **a**–**c** ROS test by DCFH-DA in 4T1 cells for control with MW irradiation, CMMP, and CMMP with MW irradiation. **d**, **e**, **g**, **h** O_2_ production measured by RDPP in 4T1 cells for CMMP with the concentration of 0, 50, 100, and 200 μg/mL. Semi-quantitative analysis of fluorescence intensity of **f** DCF in 4T1 cells for control group with MW irradiation, CMMP, and CMMP with MW irradiation and **i** RDPP in 4T1 cells for CMMP with the concentration of 0, 50, 100, and 200 μg/mL. *** indicates p < 0.001

It is known that increased ROS inside solid tumors not only promotes tumor metastasis by injuring and penetrating host tissues but also hinders the activation of the immune system. The H_2_O_2_ was the main component of ROS in tumor with a concentration of 100 × 10^–6^ M. Therefore, we examined the modulation of ROS by incubating CMMP with exogenous H_2_O_2_ for 2 h. DCFH-DA was then preincubated for 30 min before irradiation of cells with MW at 0.9 W for 5 min. The MW group exhibits obvious green fluorescence with the addition of H_2_O_2_ (Fig. 4a). We found that the green fluorescence was dramatically decreased in the presence of CMMP (100 μg/mL), suggesting high activity for scavenging H_2_O_2_. The additional MW irradiation led to the disappearance of the green fluorescence (Fig. 4f), indicating that MW enhanced the ROS scavenging capability of CMMP. It is agreed with the flow cytometry test (Additional file 1: Fig. S8).

The effect of CMMP on the production of O_2_ was evaluated by conducting an O_2_ prober [Ru(dpp)_3_]Cl_2_ (RDPP), of which the red fluorescence would be weakened or even quenched once exposed to O_2_. The 4T1 cells were preincubated with CoCl_2_ for 12 h to create hypoxia environment and were further incubated with RDPP and CMMP at different concentrations. As shown in Fig. 4d–h, significant red fluorescence was observed in the control group, indicating that the cells were in a hypoxic state. The red fluorescence decreased significantly in the presence of CMMP at 50 μg/mL. As increasing the concentration to 100 and 200 μg/mL, the red fluorescence was not observed (Fig. 4i), indicating that a large amount of intracellular O_2_ produced by CMMP is favorable for overcoming tumor hypoxia. The group in the presence of CMMP shows a lower fluorescence intensity compared with the group with CMP (Additional file 1: Fig. S9), indicating the better performance of CMMP.

For the GSH scavenge in vitro, 4T1 cells were co-incubated with CMMP and then the cells lysate were collected. The supernatant was extracted and incubated with DTNB, and the UV absorption was tested. The results were shown in Additional file 1: Fig. S10. The GSH content of the group with 100 μg/mL CMMP was less than 1/5 of that of the control group, which proved the GSH clearance function of the materials in cells.

### ICD induction and TAMs repolarization in vitro

**Fig. 5:**
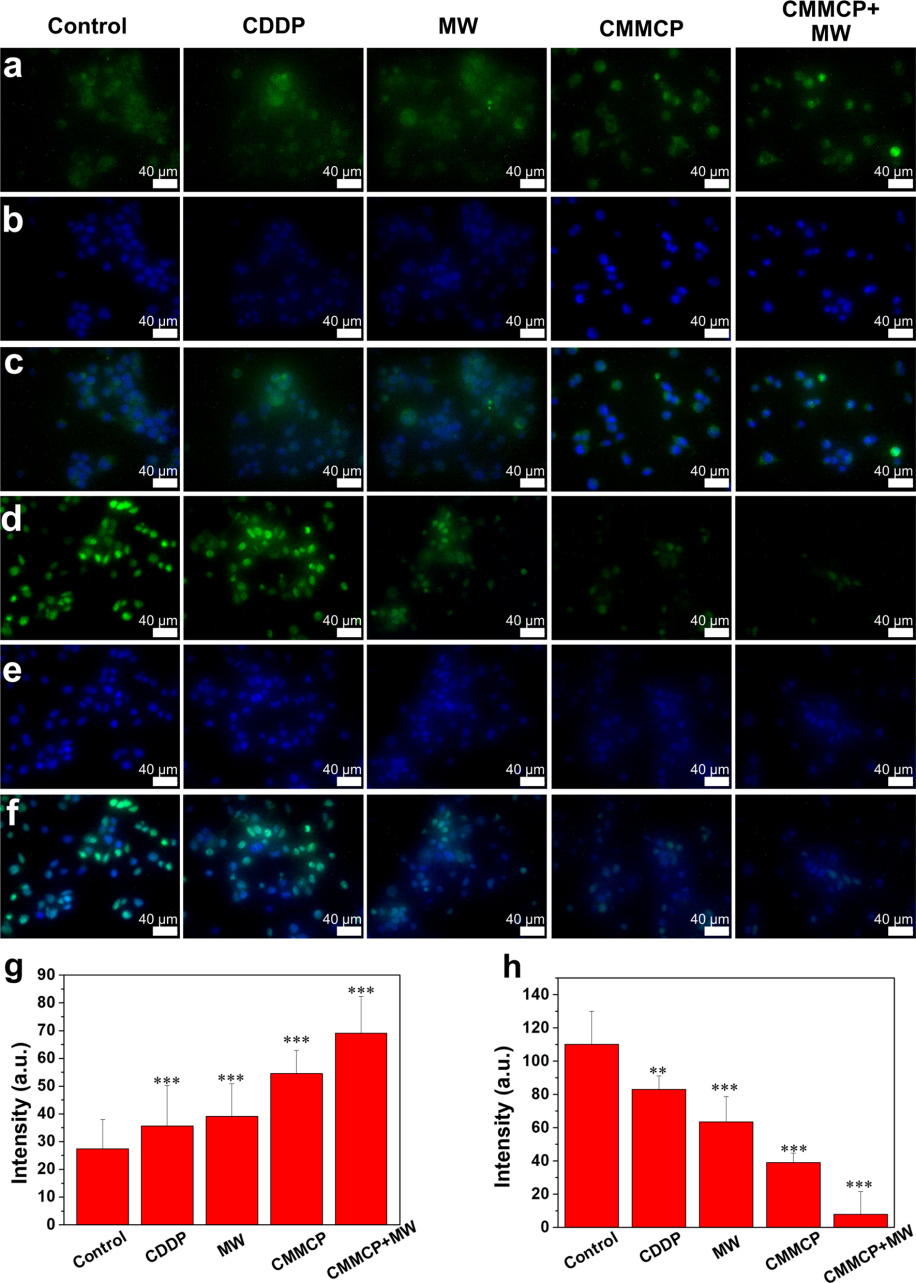
Immunofluorescence of ICD in different groups. **a** Immunofluorescence images of CRT translocated to the surface of 4T1 cells after treatment with CDDP, MW, CMMCP and CMMCP + MW. **c** Merged images for CRT and DAPI. **d** Immunofluorescence images of HMGB1 released from 4T1 after treatment with CDDP, MW, CMMCP and CMMCP + MW. **b**, **e** Cell nucleus corresponding to **a**, **d** stained with DAPI, respectively. **f** Merged images for HMGB1 and DAPI. **g**, **h** Semi-quantitative analysis of immunofluorescence intensity of CRT and HMGB1. ** indicates *p* < 0.01, *** indicates *p* < 0.001

Generally, chemotherapy or thermal therapy (photothermal therapy and magnetic hyperthermia) often induces ICD, characterized by an extracellular release of HMGB1 and cell surface of expression of CRT as antigen to promote DC maturation and activate T cell-based immunity [48, 49]. However, there were few reports about the induction of ICD by MW. Therefore, we examined the ability of CMMP (100 μg/mL) that combined MW hyperthermia with CDDP to induce CRT and HMGB1 by immunofluorescence staining. After treatment, the cells were stained with 4′,6-diamidino-2-phenylindole (DAPI). As shown in Fig. 5, we found that the expression of CRT signal in the control group was weak (Fig. 5a), and obvious green fluorescence was found after the treatment of free CDDP and MW irradiation, indicating that the thermal effect produced by MW and the free drug could also induce CRT exposure. In addition, CMM can stimulate the expression of CRT owing to the elimination of excessive ROS and GSH depletion in tumor cells, providing a favorable environment for the infiltration of immune cells. Notably, the green fluorescence was much brighter in the group of CMMCP + MW (100 μg/mL) and twofold higher than that of the control group, indicating the combination effect of CMMP, the released CDDP and MW.

MW could induce an extracellular release of HMGB1 for that of free CDDP. More reduced expression of HMGB1 treated with the combination of CMMCP and MW indicated that a large amount of HMGB1 was released to the extracellular environments. These results demonstrated that CMMP reshaped the TME to enhance the ICD induction induced by the combination effect of MW and CDDP (Fig. 5d).

**Fig. 6:**
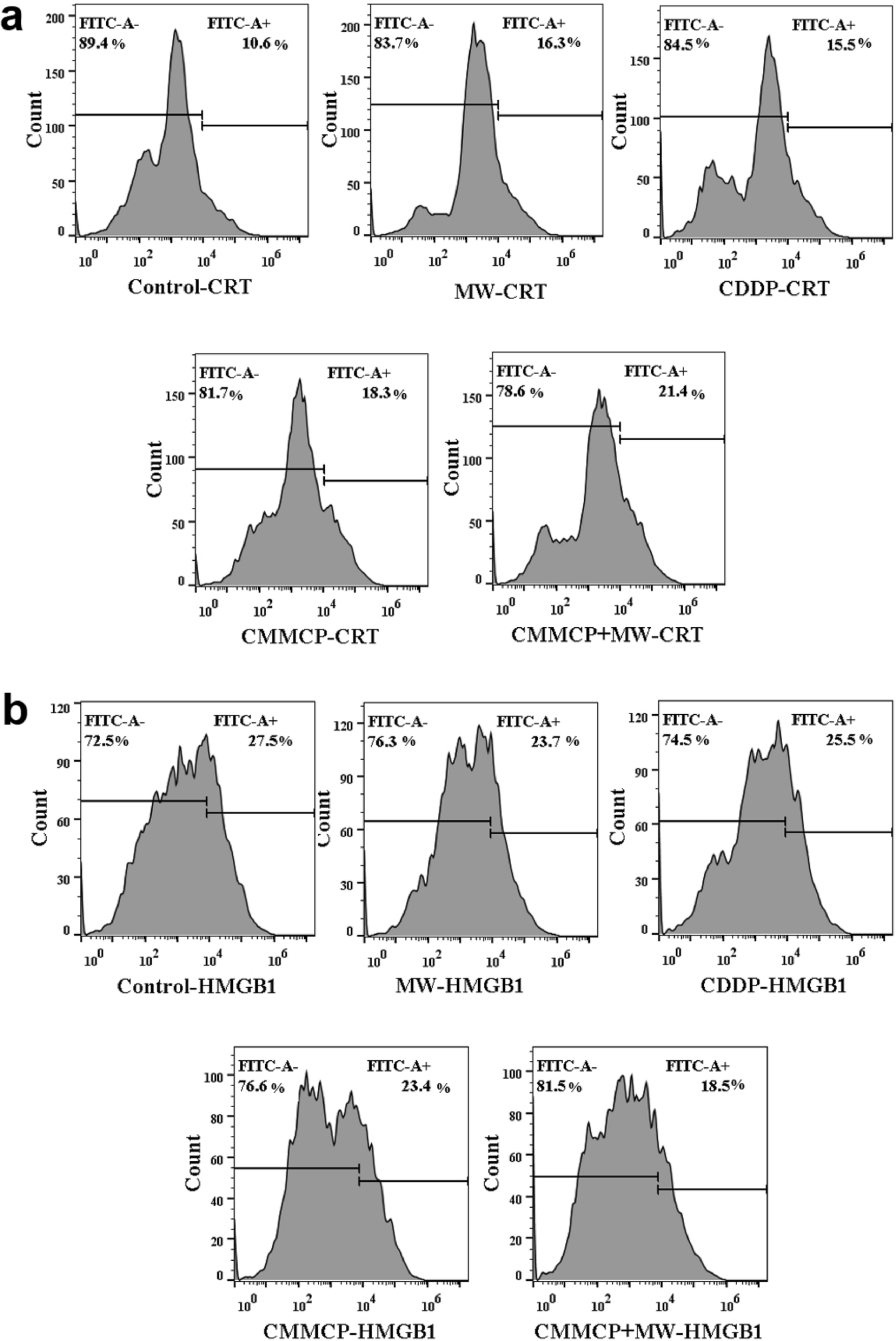
Flow cytometry analyses of **a** CRT and **b** HMGB1 of 4T1 cells after incubating with different concentration of CMMCP and CDDP with or without MW for 24 h

The fluorescence intensity was quantitatively detected by flow cytometry (Fig. 6). CMMCP + MW (100 μg/mL) treatment could stimulate the expression of CRT and the release of HMGB 1. Furthermore, the level of CRT and HMGB1 is higher than that of the MW and CDDP groups. The trend of fluorescence intensity was consistent with that of immunofluorescence measurement, further validating that CMMP was a superior adjutant to reshape the TME and enhance the induction of ICD by the CDDP and MW.

**Fig. 7:**
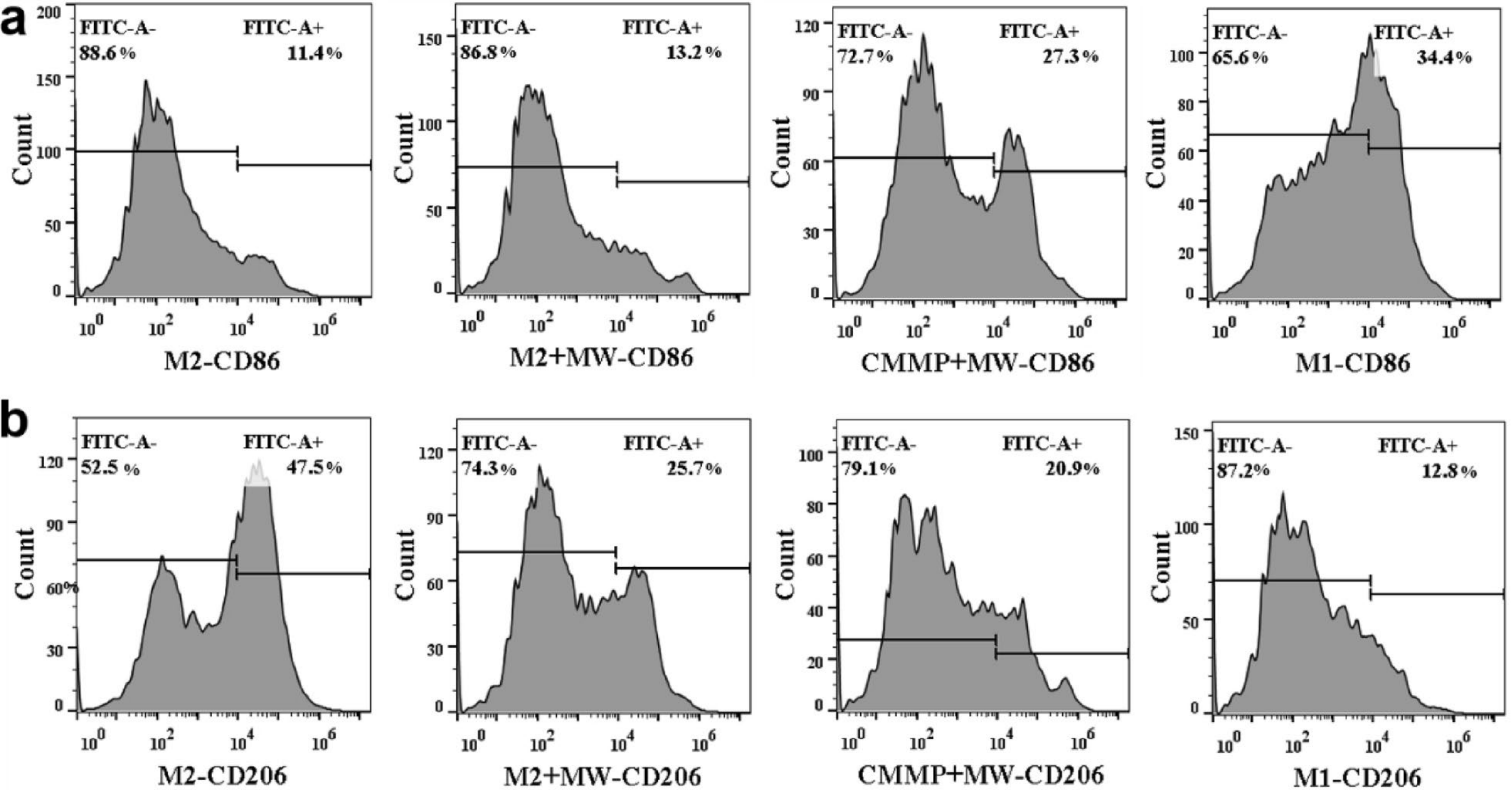
Flow cytometry analyses of CD86 and CD206 after different treatment. Receptors CD86 were represented M1 macrophages and CD206 was M2 macrophages

As an abundant component in TME, M2-type TAMs play a crucial role in promoting tumor growth. Therefore, the therapeutic efficiency can be effectively improved by the repolarization of M2-TAMs into M1-TAMs. Increasing evidence showed that ROS stimulates M2 polarization by the activation of ERK signaling and inhibits the expression of M1-related genes and proteins. CD86 and CD206 are the specific marker of M1 and M2, respectively. To evaluate the effect of CMMP on the polarization of macrophages, RAW264.7 cells were pretreated with LPS or IL-4 to differentiate M1 or M2. The M2 cells treated with MW with CMMP (100 μg/mL) or without CMMP were then stained with FITC-CD86 or FITC-CD206 and analyzed with flow cytometry. As shown in Fig. 7, the fluorescence intensity of cell marker CD86 was 34.4% in M1 macrophages, and only 12.8% fluorescence intensity of CD206 for M1 macrophages, indicating that it was successful to induce RAW 264.7 cells into M1-type polarization. After the stimulation of IL-4, the successful polarization of M2 macrophages shows obvious fluorescence for the cell marker of CD206 (11.4%) and CD86 (47.5%). Compared with the control group, the expression of the CD86 signal indicates M1 cells were increased, while that of the M2 cell marker of CD206 is reduced in the MW group. This suggest that M2 cells were partly repolarized to M1 macrophages. Notably, after treating M2 macrophages with CMMP and MW, most M2-type macrophages were transformed into M1-type macrophages, as indicated by the decreased CD206 signal of 20.9% and enhanced CD86 signal of 27.3%. This value is close to that for M1 macrophages, indicating that CMMP can effectively repolarize pro-tumor M2-type macrophages into anti-tumor M1-type macrophages under MW irradiation due to the reshaped TME.

### Biosafety and antitumor efficiency of CMMP in vivo

**Fig. 8:**
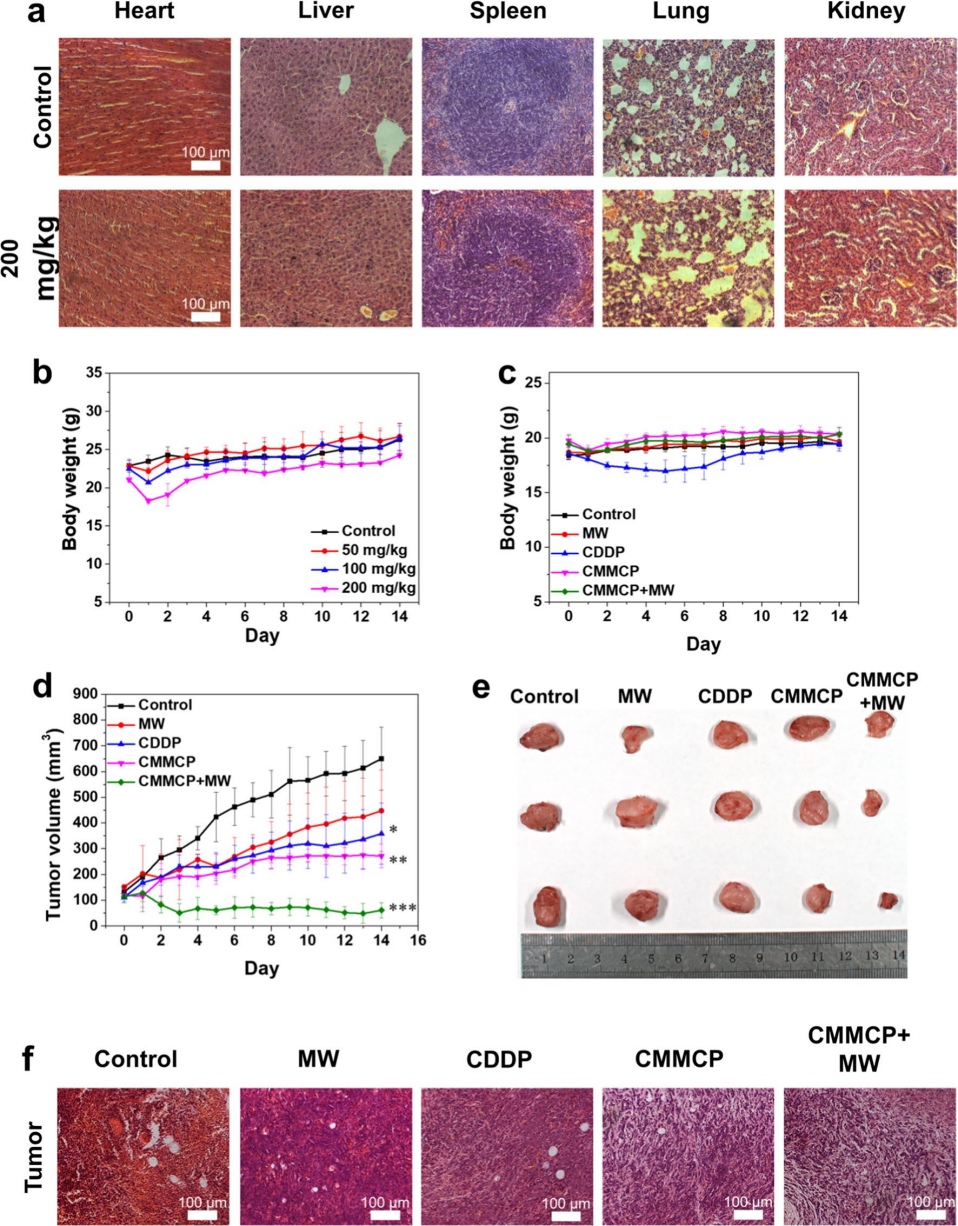
Biocompatibility and antitumor activity of CMMCP in vivo. **a** H&E staining sections of heart, liver, spleen, lung, kidney from mice injected CMMP intravenously at 200 mg/kg and control group. **b** Body weight changes of mice with injection of CMMP at different doses (0, 50, 100, and 200 mg/kg) within 14 days. **c** Body weight changes of mice from the control group, MW, CDDP, CMMCP and CMMCP + MW groups during the 14-day treatment period. **d** Tumor volume growth curve in each experimental group for 14 days. **e** Images of tumors in different groups after 14 days. **f** H&E staining sections of tumor slices for different groups killed at 14 days. * indicates *p* < 0.05, ** indicates *p* < 0.01, *** indicates *p* < 0.001

The acute toxicity in vivo was also examined to ensure biocompatibility. Healthy Balb/c mice were intravenously injected with different concentrations of CMMP. The body weight was recorded. At the end of the experiment, the blood and main organs were collected for further analysis. As shown in Fig. 8a and Additional file 1: Fig. S11, Hematoxylin and Eosin (H&E)-staining results showed that there was no significant damage to the heart, liver, spleen, lung and kidney of mice even at 200 mg/kg. The blood routine in Additional file 1: Fig. S12 also showed less abnormality. The body weight shows no obvious change. Furthermore, in comparison to the control group, no significant abnormality was observed in the detected indexes in the treated groups. Taken together, CMMP exhibits good biocompatibility at the given dosages for the potential clinical application.

To evaluate the treatment effect of CMMCP, the 4T1 tumor-bearing mice were randomly divided into 5 groups: control, MW, CDDP, CMMCP, and CMMCP + MW groups. CMMCP was injected via vein at 100 mg/kg and the corresponding concentration of CDDP was 12.3 mg/kg. MW group was subjected to 0.9 W for 5 min at 433 MHz at 6 h of post-injection. The tumor volume and body weight were recorded every day. Tumors were dissected and photographed at the end of the treatment. As shown in Fig. 8c, the body weight of mice did not change significantly after the injection of CMMCP and the MW irradiation, while the CDDP group showed a lower value than other groups before day 8, indicating that controllable release of CMMCP decreased the toxicity of free CDDP. The tumor volume curves displayed the slight inhibition effect of tumor for MW group, owing to the thermal effect caused by MW irradiation. The tumor inhibition effect and the images of bearing 4T1 tumor mice at 0 and 14 days after treatment showed that MW and CDDP group was inferior to that of the group treated with CMMCP, suggesting that the reprogrammed TME contributes to the improved anticancer effect. Notably, compared with the groups treated with CDDP, CMMP, and MW, the CMMCP + MW group displayed a higher tumor inhibition effect, confirming the greatly improved treatment efficiency due to the combined thermal treatment and immunity (Fig. 8 and Additional file 1: Fig. S13). Taken together, the CMMCP mediated combination of MW thermotherapy and immunotherapy showed excellent antitumor performance due to improved ROS scavenging, O_2_ supply, and GSH depletion.

### Conversion of cold tumors to hot

**Fig. 9:**
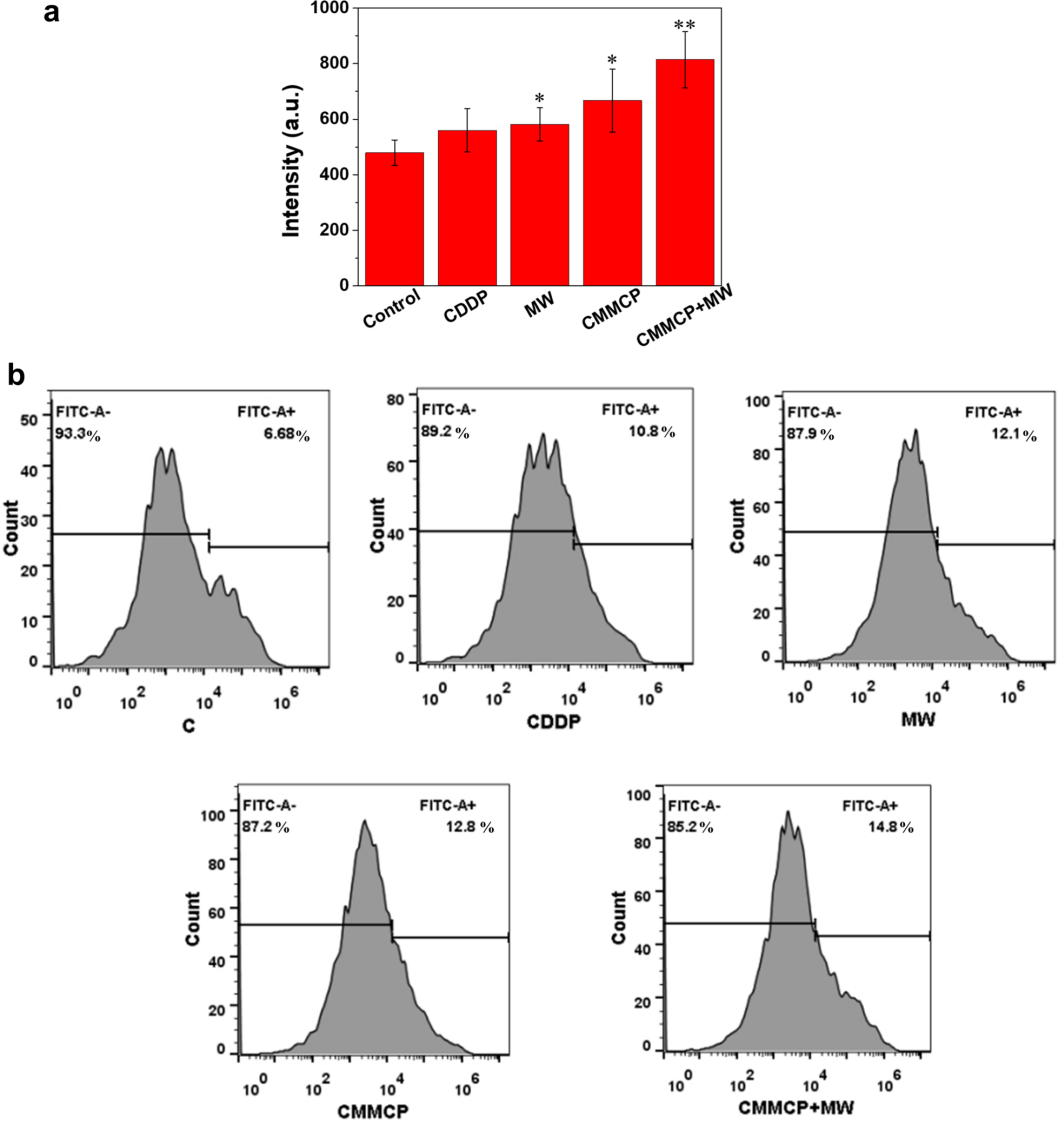
Immune activation in vivo. Quantitative analysis of CTL infiltration for **a** spleens at 14 days and **b** tumors 24 h after different treatment. CD8^+^ T cells was analyzed flow cytometry. * indicates *p* < 0.05, ** indicates *p* < 0.01

Encouraged by the excellent performance, the immune effect was investigated on day 14 by analysis of the expansion of CD8^+^ T cells. The spleens were collected from each group to test the infiltration of CD8^+^ T cells via flow cytometry. Figure 9a showed the ratio of CD8^+^ T cells was increased after treated with CMMCP, and the ratio of CD8^+^ T cells was even higher in the group combined CMMCP with MW irradiation, the results are consistent with the fluorescence intensity of ICD, reflecting the ability of CMMCP to enhance the infiltration and activation of CD8^+^ T cells. The infiltration of CD8^+^ T cells in the tumor region 24 h after MW treatment was also evaluated (Fig. 9b). It could be observed that CMMCP + MW led to more cytotoxic CD8^+^ T cells than other treated groups, indicating the improvement of the amount and function of CD8^+^ T cells for inhibiting tumor growth.

## Conclusion

In summary, we designed a novel microwave-chemo-immunostimulant of CMMCP for reversing TME. The Ce–Mn MOF particles can scavenge ROS through reversible Ce^3+^ and Ce^4+^ redox reaction. The Mn-doping and MW are favorable for the elimination of ROS for rebuilding redox homeostasis. Thus, the excessive ROS in the tumor could be converted into O_2_ to impair the generation of ROS induced by hypoxia. In addition, we find that the robust ICD was reserved by a combination of MW hyperthermia and chemotherapy enabled by CDDP in the reprogramming of the redox microenvironment. Furthermore, the M2 type of TAM was found to be effectively reversed into M1 macrophages. The in vivo experiments show that the combined regulation of ICD and TAM can activate the immune system to produce stronger CD8^+^ T cell infiltration, resulting in reversed cold tumors and the enhanced combination of MW therapy and immunotherapy. Our findings with CMMCP reveal that the ROS scavenging combined with GSH depletion and O_2_ production can benefit the optimization of tumor microenvironment and achieve the synergistic treatment of tumor by immunotherapy and MW hyperthermia, which presents a promising and gentle therapeutic approach in cancer treatment.

## Supplementary Information

**Additional file 1: Fig. S1**. SEM image of CM (**a**). TEM images of CMMCP after 6 h degradation in pH 5.7 and 7.4 solution (**b**–**c**). **Fig. S2**. The high-resolution Pt 4f XPS spectrum of CMMCP after Ar^+^ etching (**a**) and CDDP (**b**). The high-resolution Mn2p XPS spectrum of CMMCP (**c**). **Fig. S3**. Standard curves of (**a**) GSH and (**b**–**d**) CDDP in DMF, pH 5.7 and 7.4 solution measured by UV absorption. **Fig. S4**. ROS scavenging ability for CMM and CMMP at 0.4, 1.2 and 2 mg/mL with or without MW irradiation.**Fig. S5**. (**a**) GSH clearance rate for CMMP at 0.16, 0.48, 0.8 and 1.6 mg/mL. **Fig. S6**. O_2_ production for (**a**) CM and (**b**) CMM at 0.5, 1.5, 2.5, 5 mg/mL. **Fig. S7**. Hyperspectral images for 4T1 cells treated without CMMP. Biocompatibility for CMM and CMMP treated with 4T1 (**a**) and L929 cells (**b**). **Fig. S8**. ROS test in 4T1 cells incubated with CMP and CMMP with MW at 100 μg/mL measured by flow cytometry test. **Fig. S9**. RDPP test in 4T1 cells treated with CMP and CMMP at 100 μg/mL measured by flow cytometry test. **Fig. S10**. GSH content in 4T1 cells co-incubated with 100 μg/mL CMMP for 12 h. *** indicates *p* < 0.001. **Fig. S11**. Heart, liver, spleen, lung, and kidney of the mice treated with CMMP at 50 and 100 mg/kg and the mice from the different treated groups. **Fig. S12**. Blood routine examination including (**a**) LYM, (**b**) MCV, (**c**) RDWCV, (**d**) MON, (**e**) RDWSD, (**f**) MPV, (**g**) PLT, (**h**) HCT, and (**i**) PCT of the mice with the treatment of CMMP (0, 50, 100, and 200 mg/kg) via tail vein injection in vivo. **Fig. S13**. Photos of the bearing 4T1 tumor mice at 0 and 14 d after MW irradiation.

## Data Availability

The raw/processed data required to reproduce these findings cannot be shared at this time due to technical or time limitations.
