# Supplementary material for: Reversing the immunosuppressive microenvironment with reduced redox level by microwave-chemo-immunostimulant Ce–Mn MOF for improved immunotherapy
Source: J Nanobiotechnology. 2022 Dec 3;20:512. doi: 10.1186/s12951-022-01699-w (PMC9719648; doi:10.1186/s12951-022-01699-w)
Supplement: Supplementary file 1 — Additional file 1: Fig. S1. SEM image of CM (a). TEM images of CMMCP after 6 h degradation in pH 5.7 and 7.4 solution (b–c). Fig. S2. The high-resolution Pt 4f XPS spectrum of CMMCP after Ar+ etching (a) and CDDP (b). The high-resolution Mn2p XPS spectrum of CMMCP (c). Fig. S3. Standard curves of (a) GSH and (b–d) CDDP in DMF, pH 5.7 and 7.4 solution measured by UV absorption. Fig. S4. ROS scavenging ability for CMM and CMMP at 0.4, 1.2 and 2 mg/mL with or without MW irradiation.Fig. S5. (a) GSH clearance rate for CMMP at 0.16, 0.48, 0.8 and 1.6 mg/mL. Fig. S6. O2 production for (a) CM and (b) CMM at 0.5, 1.5, 2.5, 5 mg/mL. Fig. S7. Hyperspectral images for 4T1 cells treated without CMMP. Biocompatibility for CMM and CMMP treated with 4T1 (a) and L929 cells (b). Fig. S8. ROS test in 4T1 cells incubated with CMP and CMMP with MW at 100 μg/mL measured by flow cytometry test. Fig. S9. RDPP test in 4T1 cells treated with CMP and CMMP at 100 μg/mL measured by flow cytometry test. Fig. S10. GSH content in 4T1 cells co-incubated with 100 μg/mL CMMP for 12 h. *** indicates p < 0.001. Fig. S11. Heart, liver, spleen, lung, and kidney of the mice treated with CMMP at 50 and 100 mg/kg and the mice from the different treated groups. Fig. S12. Blood routine examination including (a) LYM, (b) MCV, (c) RDWCV, (d) MON, (e) RDWSD, (f) MPV, (g) PLT, (h) HCT, and (i) PCT of the mice with the treatment of CMMP (0, 50, 100, and 200 mg/kg) via tail vein injection in vivo. Fig. S13. Photos of the bearing 4T1 tumor mice at 0 and 14 d after MW irradiation. [file 12951_2022_1699_MOESM1_ESM.docx]

Reversing the Immunosuppressive Microenvironment with Reduced Redox Level by Microwave-Chemo-Immunostimulant Ce-Mn MOF for Improved Immunotherapy

Zhiheng Zeng,^†,‡^ Changhui Fu,^†,*^ Xiaohan Sun,^ǂ^ Meng Niu, ^ǂ^ Xiangling Ren,^†^ Longfei Tan, ^†^ Qiong Wu, ^†^ Zhongbing Huang, ^‡,*^ Xianwei Meng^†,*^

†Laboratory of Controllable Preparation and Application of Nanomaterials, Technical Institute of Physics and Chemistry, Chinese Academy of Sciences, Beijing 100190, China

CAS Key Laboratory of Cryogenics, Technical Institute of Physics and Chemistry, Beijing 100190, China

E-mail: [mengxw@mail.ipc.ac.cn](mailto:mengxw@mail.ipc.ac.cn), [fuchanghui@mail.ipc.ac.cn](mailto:fuchanghui@mail.ipc.ac.cn)

‡College of Biomedical Engineering, Sichuan University, Chengdu 610065 China

E-mail: [zbhuang@scu.edu.cn](mailto:zbhuang@scu.edu.cn)

ǂDepartment of Interventional Radiology, The First Hospital of China Medical University, Shenyang 110000, China

**Experimental section**

**Materials**

Ammonium cerium nitrate (Ce(NH_4_)_2_(NO_3_)_6_) (99.99%), 2-amino-terephthalic acid (H_2_BDC-NH_2_) (98%), and dopamine hydrochloride (PDA) (98%) were purchased from J&K Scientific Co., Ltd. Manganese chloride tetrahydrate (MnCl_2_·4H_2_O) (99.0%) was purchased from Macklin Biochemical Technology Co., Ltd. Polyvinylpyrrolidone K30 (PVP) (99.5%) was purchased from Beijing Chemical Reagent Company. Cisplatin (CDDP) was purchased from Beijing Huafeng Lianbo Technology Co., Ltd. Methanol (99.5%) and N, N-Dimethyl formamide (99.5%) were purchased from Modern Oriental Technology Development Co., Ltd. Calreticulin DAPI, DCFH-DA, Rabbit Monoclonal Antibody, HMGB1 Rabbit Monoclonal Antibody, CD86 Rabbit Monoclonal Antibody, and Alexa Fluor 488 IgG were purchased from Shanghai Beyotime Biotechnology Co., Ltd. CD206 Polyclonal antibody was purchased from Proteintech.

**Methods**

**Synthesis of CMM**

The CMM were prepared by the solvothermal method. 0.079 g 2-aminoterephthalic acid (H_2_BDC), 0.12 g cerium ammonium nitrate, 0.215 g manganese chloride tetrahydrate and 0.25 g polyvinylpyrrolidone (PVP-K30) were dissolved in 9 mL N, N-dimethylformamide (DMF) and 1.2 mL methanol. The mixed solution was transferred to a Teflon-lined stainless-steel autoclave and heated to 150℃ for 4 h. The precipitation was collected after washing with deionized water and ethanol respectively for three times. The obtained sample was suspended in ethanol for later use.

**Synthesis of CMMCP**

CMM and CDDP were added into 4 mL DMF, followed by shaking for 3 h at room temperature. After 3 h, the CMM-loaded CDDP (CMMC) was centrifuged and washed at 11000 rpm for 5 min. To ensure the on-demand release, a ultrathin layer of dopamine hydrochloride was coated on the surface of the obtained CMMCP. In detail, 25 μL of ammonia was added into the methanol solution of dopamine hydrochloride and CMMC at 2 mg/mL and then stirred for 2 h. The CMMC coated with dopamine hydrochloride (CMMCP) was obtained after centrifugation and stored at 4 ℃ in a dark environment.

**Stability and degradation of CMMCP**

The CMMCP nanoparticles were dispersed in PBS 5.7 and PBS 7.4 at 1 mg/mL, the dispersion underwent a water bath for 2, 6, 12 and 24 h at 37℃, respectively, and then re-dispersed in ethanol for TEM characterization.

**Drug release of CMMCP**

The same experiment conditions of degradation of CMMCP were performed for the analysis of CDDP content released from CMMCP, the supernatant was collected and measured the ultraviolet absorption at 311 nm after centrifugation. The precipitation was re-dispersed in PBS at different pH values for drug release.

**O_2_ production in the solution and in vitro**

CMM can react with hydrogen peroxide and transform into O_2_, the produced O_2_ was measured using dissolved oxygen and a BOD meter. 10 μL of 10 mM hydrogen peroxide was added into a 2 mL solution containing different concentration materials at 0.5, 1.5,2.5 and 5 mg/mL, respectively. Then, the produced O_2_ content in the solution was recorded every 30 s for 20 min.

**ROS measurement in the cell-free solution**

DCFH-DA probe was used to assess the effect of CMMP on the reduction of ROS under microwave (MW) irradiation as the ROS could react with DCFH-DA form fluorescent DCF. Specifically, 1, 3, and 5 mg materials were suspended in 2.3 mL deionized water, 5 μM diluted DCFH-DA and 10 μL 2% hydrogen peroxide were added into the solution, followed by MW irradiation at the power of 0.9 W for 5 min at 433 MHz. After 1 h reaction in the dark environment, the supernatant was collected and analyzed with a fluorescence spectrometer at the excitation of 480 nm. The decrease of fluorescence intensity with different concentrations of samples at the emission of 520 nm was used to evaluate the ROS scavenging performance of samples after MW irradiation.

**Glutathione measurement**

1, 3, 5, and 10 mg materials were immersed in GSH solution (50 μg/mL) for 5 min. 4 mL of neutral PBS and 0.4 mL of DTNB were added to the solution, followed by measuring the UV absorption at 412 nm for the supernatant.

**Cell experiments in vitro**

The cell cytotoxicity of CMM and CMMP was detected by MTT assay. 8×10^3^ cells were seeded on 96-well plates and cultured with different concentrations of materials at 0, 25, 50, 75, 100, 125, 150, and 200 μg/mL for 24 h. Then, 20 μL MTT (3-(4,5-dimethylthiazol-2-yl)-2,5-diphenyltetrazoliumbromide) at the final concentration of 5 mg/ml was added. After 4 h incubation, 150 μL DMSO was added, followed by performing the colorimetric measurement with a scanning multiwell spectrometer at 492 nm.

For tumor therapy *in vitro*, 4T1 cells were seeded on the 12-well plates and cultured overnight. The cells were divided into the control group, MW group, CDDP group, CMMCP group, and CMMCP with MW group. CMMCP at a final concentration of 100, 200, and 400 μg/mL were co-cultured with the cells for 24 h. The MW treated groups received MW irradiation with the power of 0.9 W for 5 min. After MW irradiation, 100 μL cell suspension was transferred to the 96-well plates and put back in the incubator. After cultivation for 12 h, an MTT assay was carried out to test the cell viability to evaluate the therapeutic effect of the material on the cells.

**Intracellular ROS detection**

To analyze the content of reactive oxygen species (ROS) in tumor cells treated with CMMP, 1×10^5^ 4T1 cells were seeded on the 6-well plates and cultured overnight. The cells were divided into the control group, CMMP group, and CMMP with the MW group. CMMP at a final concentration of 100 μg/mL were co-cultured with the cells for 24 h. The MW-treated group received MW irradiation with a power of 0.9 W for 5 min. Before the MW irradiation, 500 μM hydrogen peroxide was added and incubated for 2 h. Then the cells were washed with PBS and digested. After the cell suspension was treated with MW irradiation, 1 mL of 5 μM DCFH-DA was added and incubated for 30 min at 37 ℃, washed with PBS twice, and observed with a microscope.

**Intracellular O_2_ detection**

The O_2_ production *in vitro* was examined using ruthenium dichloride (RDPP), a green fluorescent prober, which would be quenched by the intracellular O_2_. In detail, 4T1 cells were inoculated into 6-well plates overnight, namely, CMMP with a concentration of 50, 100, and 200 μg/mL. Cells were incubated with 100 μg/mL of cobalt dichloride solution that was dispersed in basal culture medium (DMEM) and was added for creating a hypoxic environment. After incubation for 12 h, the cells were washed with PBS, followed by adding 10 μg/mL RDPP for another 10 h. 500 μM hydrogen peroxide was added to the plates. The fluorescence of 6-well plates was observed after being washed with neutral PBS after 2 h adding of hydrogen peroxide.

**Intracellular GSH detection**

For the GSH scavenge *in vitro,* 4T1 cells were co-incubated with 100 μg/mL CMMP for 12 h, and then the cells lysate were collected. The supernatant was extracted and incubated with DTNB (50 μL, 400 μM), and the UV absorption at 412 nm was tested after 30 min incubation.

**Fluorescence immunostaining and flow cytometry of ICD**

To analyze the effect of CMMCP (100 μg/mL) on the immunogenic cell death (ICD) signals, CRT and HMGB1 were used as representative signaling molecules. 5×10^4^ 4T1 cells were seeded on the 12-well plates. After incubating the cells with a final concentration of 100 μg/mL of CMMCP, and the corresponding concentration of CDDP for 24 h, the cells were washed with PBS. To investigate the influence of ROS on ICD signals, additional hydrogen peroxide (100 μM) was added to the 12 well plates. The group of experiments and operation steps were the same as above, only excessive (100 μM) hydrogen peroxide was added when the materials and free drugs was added. Immunofluorescence observation was performed after immunofluorescence staining was completed. The prepared cell suspension was irradiated with 433 MHz MW at 0.9 W for 5 min and then put back in the incubator. After 8 h, the cells were fixed for 10 min by the fix-solution, followed by 1.5% BSA to block for 1 h. The primary and secondary antibodies were diluted according to the manufacturer’s direction. Samples were incubated at 4℃ with diluted primary antibody (anti-CRT and anti-HMGB1) overnight. Antimouse or antirabbit antibodies conjugated with florescence were used as the secondary antibody. A diluted secondary antibody was incubated with cells for 1 h. Finally, the cell nucleus was stained with DAPI for 10 min. After each step, cells were washed three times with buffer for 5 min. The fluorescence was observed using a Nikon Ti-S microscope after adding the antifade solution. Quantitative analysis of fluorescence signals was performed with flow cytometry. The samples were prepared by the same approach of fluorescence immunostaining except for nuclear staining.

**Macrophage polarization test**

To test the ability of the CMMCP (100 μg/mL) to repolarize M2 macrophages into M1 macrophages, RAW 264.7 cells were pretreated with 100 ng/mL LPS for 24 h to induce the M1 phenotype. In the meantime, 20 ng/mL IL-4 was used to induce RAW 264.7 cells to differentiate and polarized to the M2 phenotype. Then, M2 phenotype macrophages were incubated with CMMCP at concentrations of 100 μg/mL for 24 h. The prepared cell suspension was irradiated with 433 MHz MW at 0.9 W for 5 min and then put back in the incubator. After 8 h, the cells were fixed for 10 minutes by the fix-solution, followed by 1.5% BSA was used to block for 1 h. The cells were incubated with M1-related marker anti-CD80 and M2-related marker anti-CD206. Antimouse or antirabbit antibodies conjugated with florescence were used as the secondary antibody. Diluted secondary antibodies were incubated with cells for 1 h. Finally, the cell nucleus was stained with DAPI for 10 min. After each step, cells were washed three times with buffer for 5 min. The fluorescence was observed using a Nikon Ti-S microscope after adding the antifade solution. Quantitative analysis of fluorescence signal was performed with flow cytometry. The samples were prepared by the same approach of fluorescence immunostaining except for nuclear staining.

**Endocytosis evaluation**

To observe the endocytosis of the CMMP in cells, 4T1 cells were seeded on the sterile cover glass overnight. After incubating the cells with CMMP at 100 μg/ mL for 12 h, the cover glass was washed and fixed with 1.5 mL 4% neutral formaldehyde for 15 min. Cells were washed lightly with sterile water and sealed with resin on the glass slide. The signal spectrum library of the CMMP in the solution was first established using a hyperspectral imager. Then the location of CMMP in cells could be overlaid according to the existing library.

**Animal acute toxicity test**

The BALB/c mice, female, 18-22 g, were used in this experiment, which was purchased from Vital River Laboratories Co., Ltd. Animal experiments were carried out strictly in accordance with the local ethics committee. Mice was raised at 25±3℃ and 50-55% humidity. BALB/c mice were injected with CMMP through the tail vein at 50, 100, and 200 mg/kg (n=3), respectively. Clinical manifestation and their weight was recorded. Blood was collected for the analysis of blood biochemical and routine after 14 d. Heart, liver, spleen, lung, and kidney from different groups of mice were soaked in 4% neutral formaldehyde, and then tissue samples were sectioned, dehydrated, and embedded according to the standard protocol. The tissue slices were stained with hematoxylin and eosin (H&E) and observed with a Nikon Ti-S microscope.

**Anti-tumoral experiment *in vivo***

The anti-tumor effect of CMMCP *in vivo* experiments was performed using the 4T1 tumor model. The tumor was inoculated with 200 μL at the cell concentration of 10^6^. When the tumor reached about 150 mm^3^, BALB/c mice were divided into five groups: control group, MW group, CDDP alone group, CMMCP alone group, and CMMCP plus MW group (n=5). After intravenous injection of CMMCP at 100 mg/kg for 6 h, the mice were irradiated with MW at 0.9 W for 5 min. During the whole experiment, the mice were weighed and the tumor size was measured every two days. On the 14^th^ day of therapy, all the mice were killed, and the tumors were dissected and weighted. Heart, liver, spleen, lung, and kidney from different groups of mice were soaked in 4% neutral formaldehyde, and then tissue samples were sectioned, dehydrated, and embedded according to the standard protocol. The tissue slices were stained with hematoxylin and eosin (H&E) and observed with a Nikon Ti-S microscope.

**
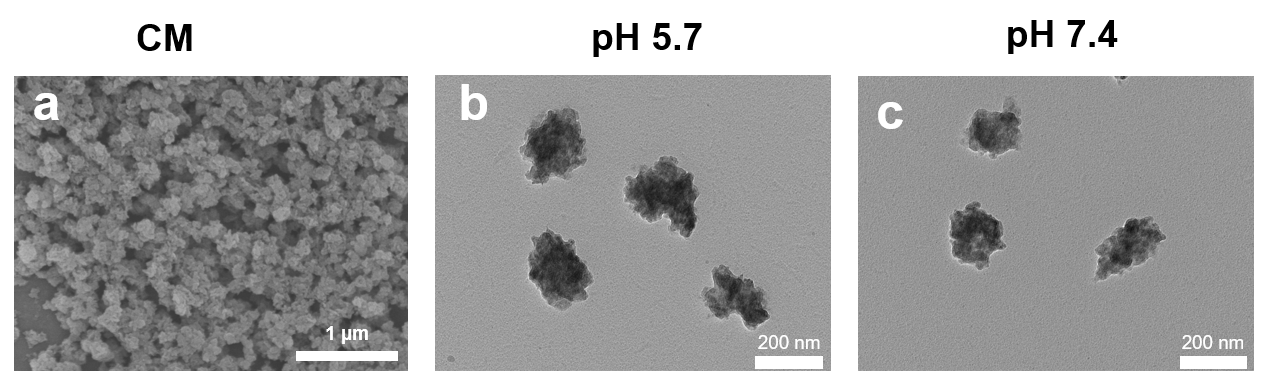
**Fig. S1 SEM image of CM (a). TEM images of CMMCP after 6 h degradation in pH 5.7 and 7.4 solution (b-c).


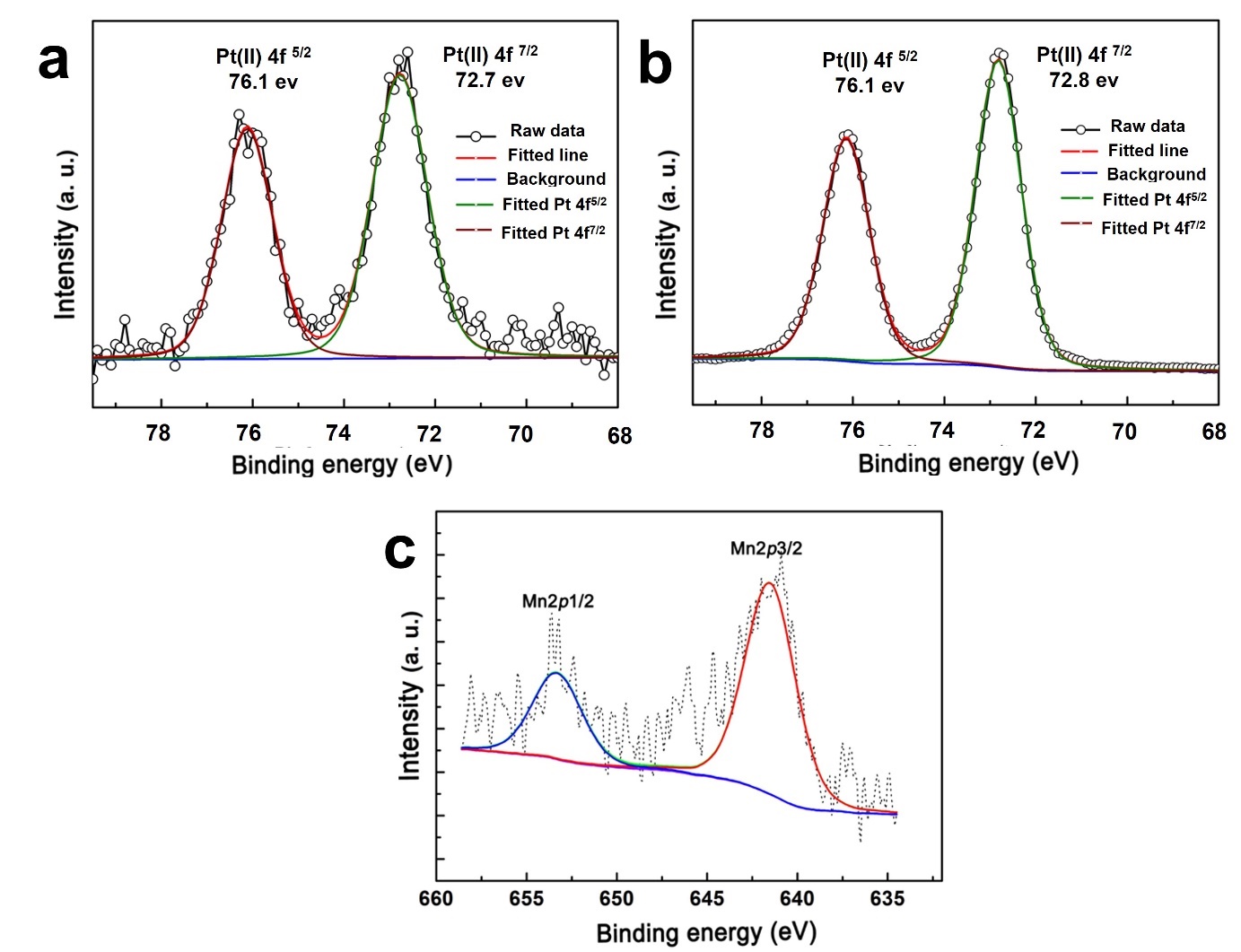


Fig. S2 The high-resolution Pt 4f XPS spectrum of CMMCP after Ar^+^ etching (a) and CDDP (b). The high-resolution Mn2p XPS spectrum of CMMCP (c).

*
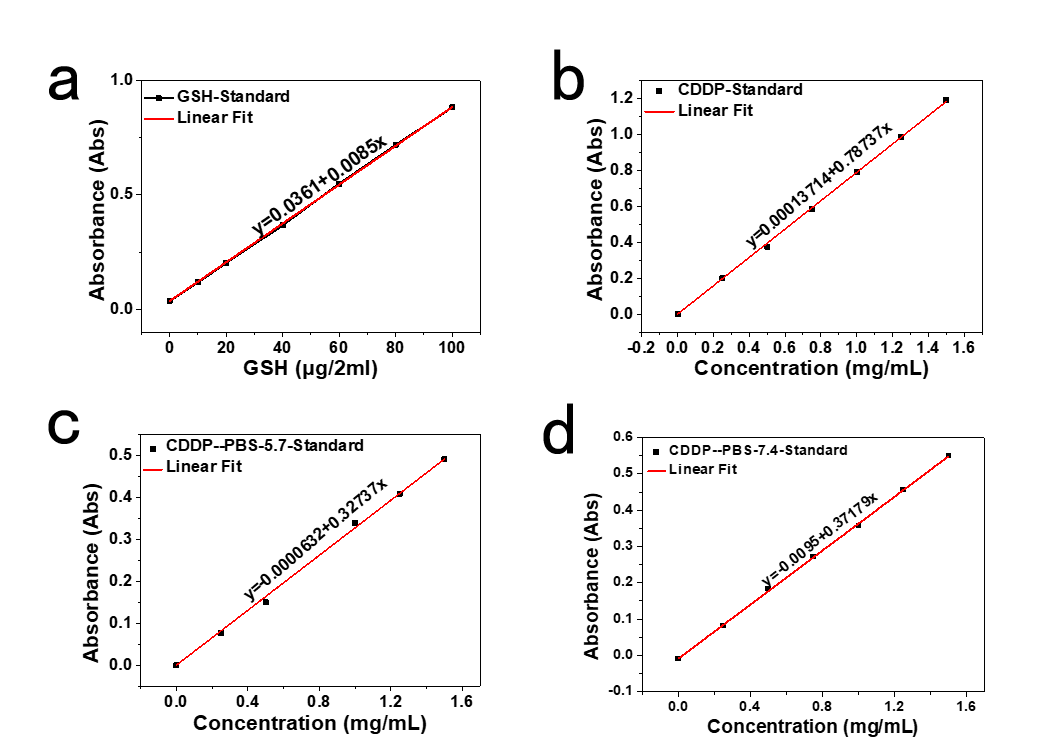
*

Fig. S3 Standard curves of (a) GSH and (b-d) CDDP in DMF, pH 5.7 and 7.4 solution measured by UV absorption.

*
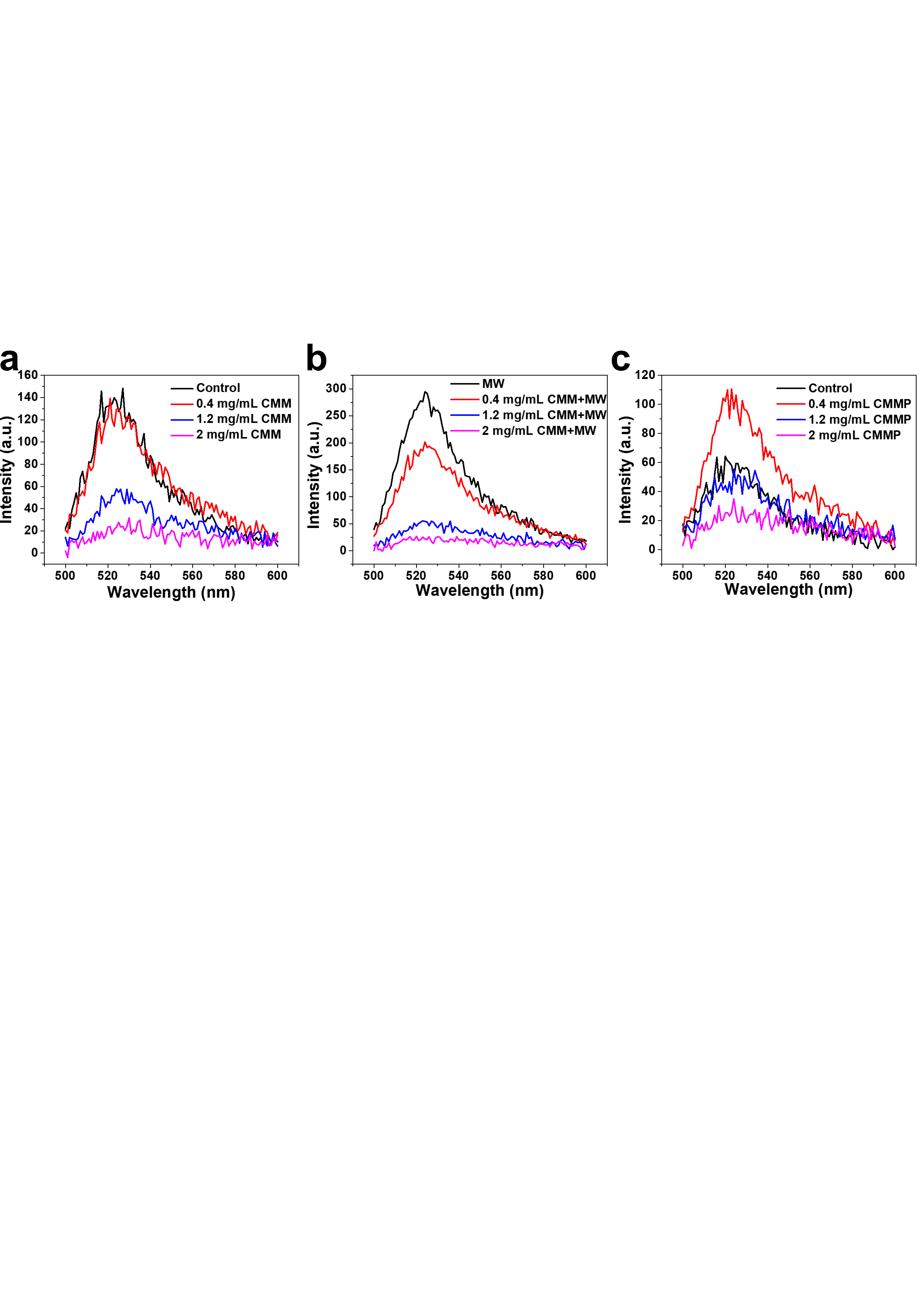
*

Fig. S4 ROS scavenging ability for CMM and CMMP at 0.4, 1.2 and 2 mg/mL with or without MW irradiation.

**
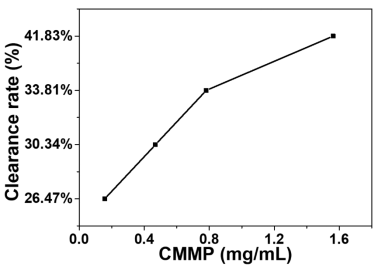
**

Fig. S5 (a) GSH clearance rate for CMMP at 0.16, 0.48, 0.8 and 1.6 mg/mL.

*
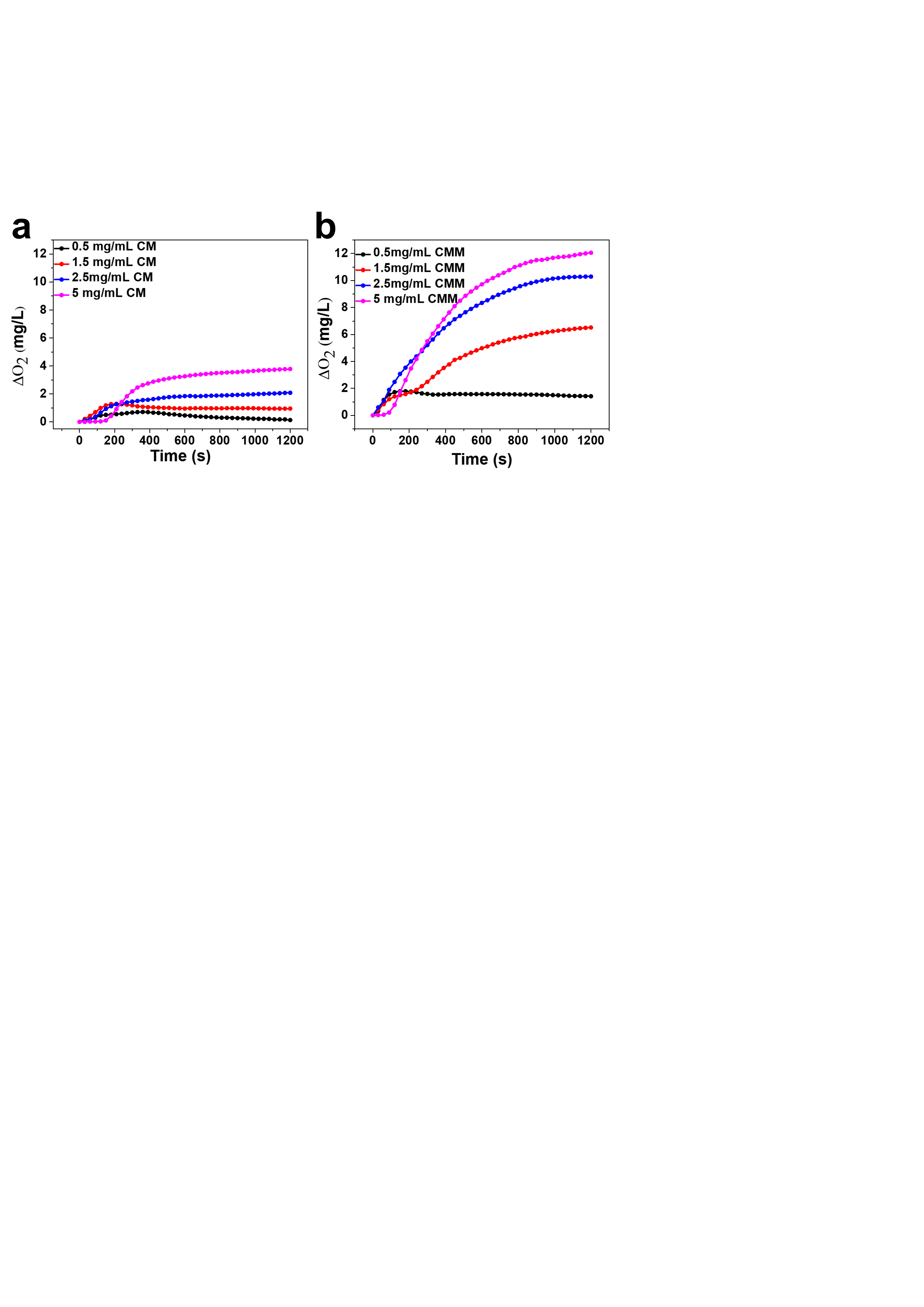
*

Fig. S6 O_2_ production for (a) CM and (b) CMM at 0.5, 1.5, 2.5, 5 mg/mL.


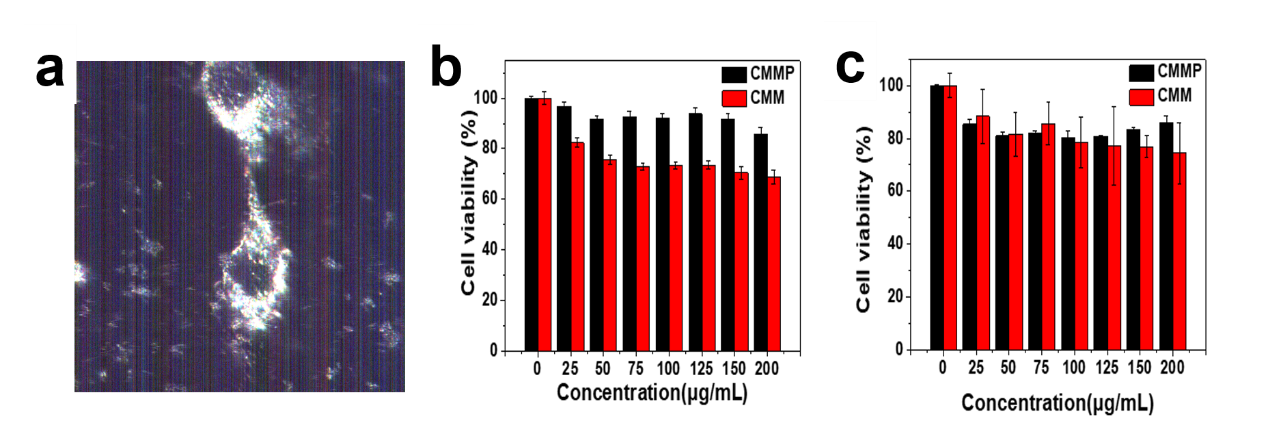


Fig. S7 Hyperspectral images for 4T1 cells treated without CMMP. Biocompatibility for CMM and CMMP treated with 4T1 (a) and L929 cells (b).


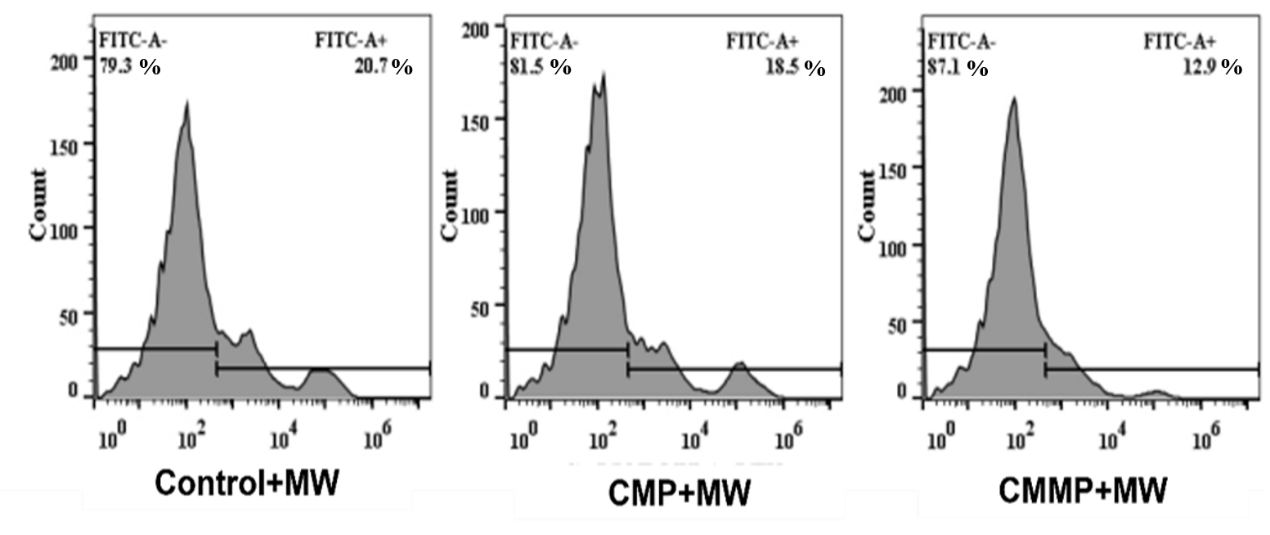


Fig. S8 ROS test in 4T1 cells incubated with CMP and CMMP with MW at 100 μg/mL measured by flow cytometry test.

**.**


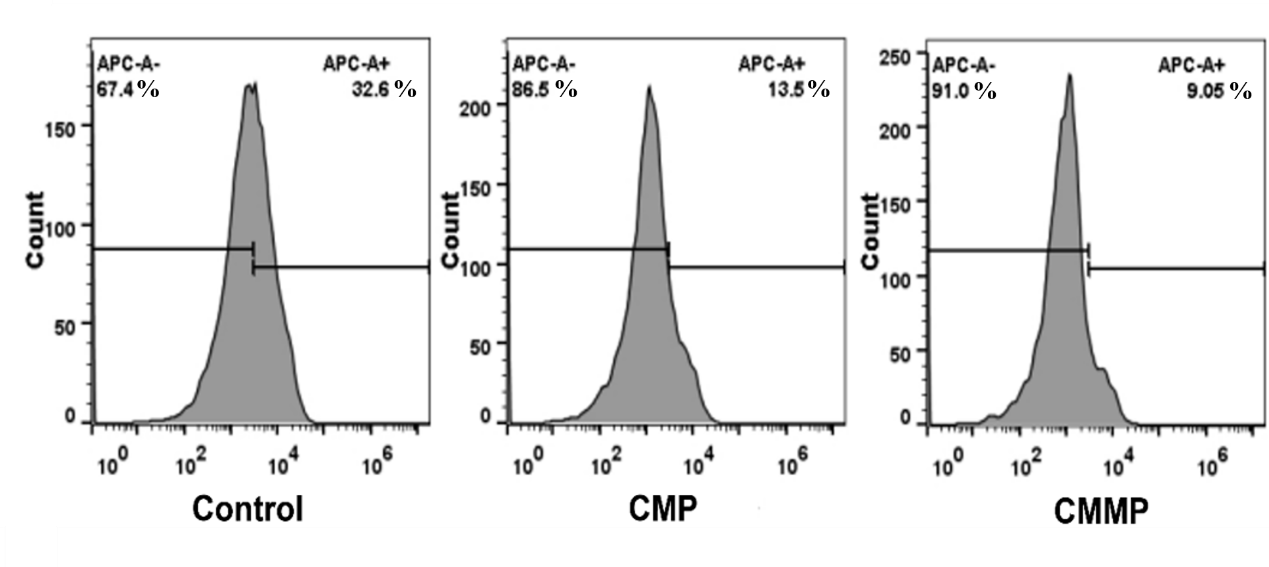


Fig. S9 RDPP test in 4T1 cells treated with CMP and CMMP at 100 μg/mL measured by flow cytometry test.

**
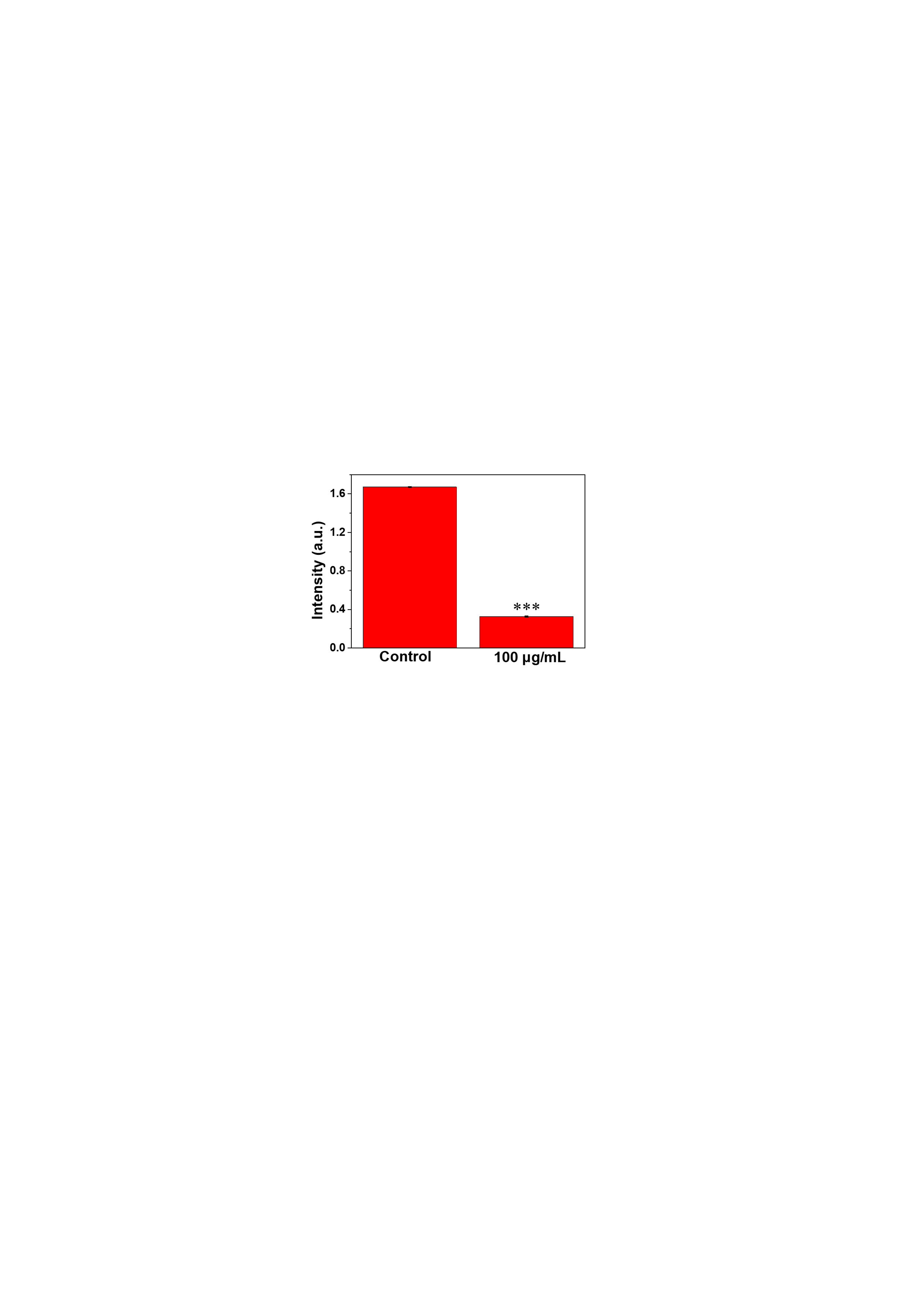
**

Fig. S10 GSH content in 4T1 cells co-incubated with 100 μg/mL CMMP for 12 h. *** indicates *p* < 0.001.

*
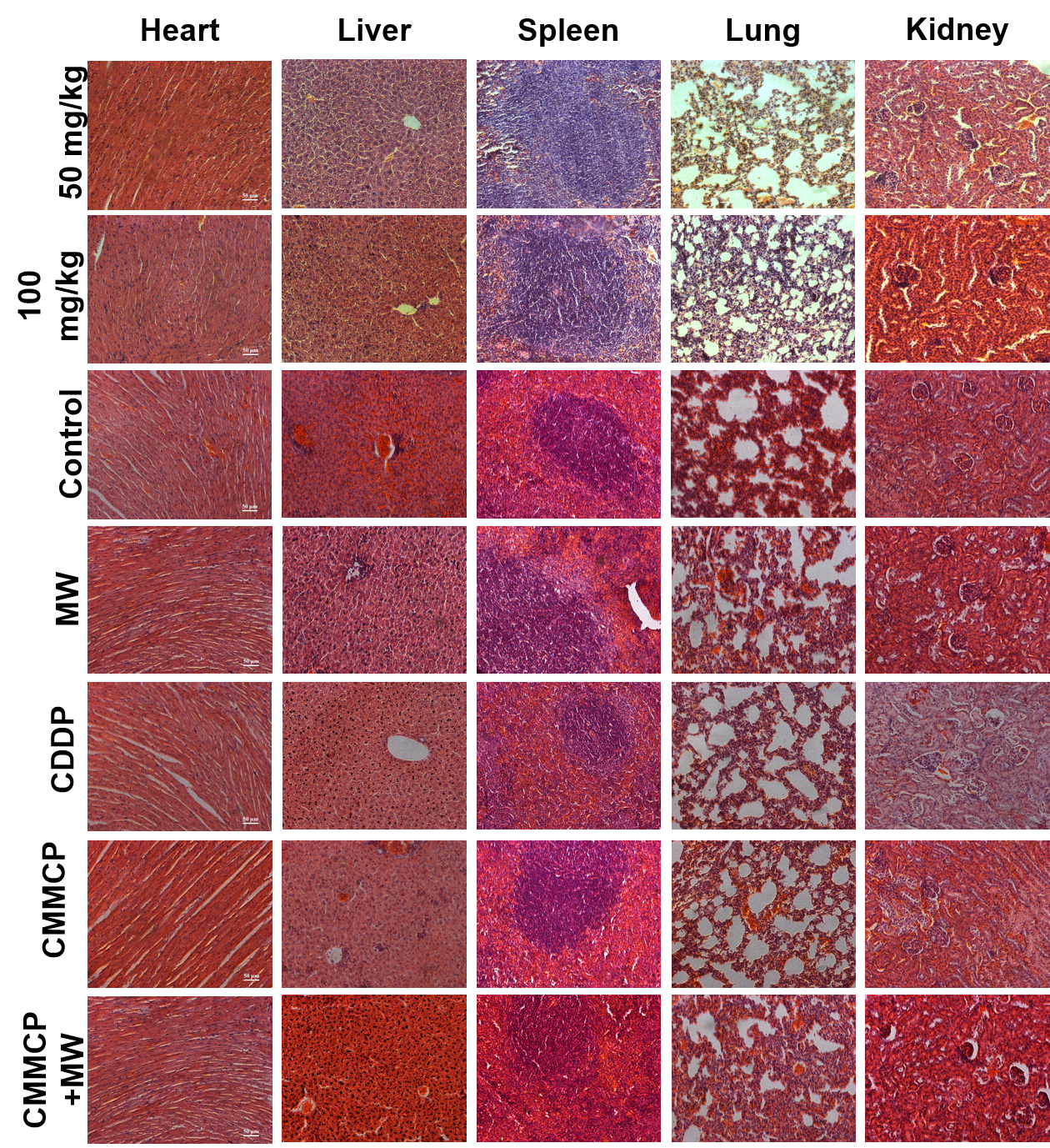
*

Fig. S11 Heart, liver, spleen, lung, and kidney of the mice treated with CMMP at 50 and 100 mg/kg and the mice from the different treated groups.


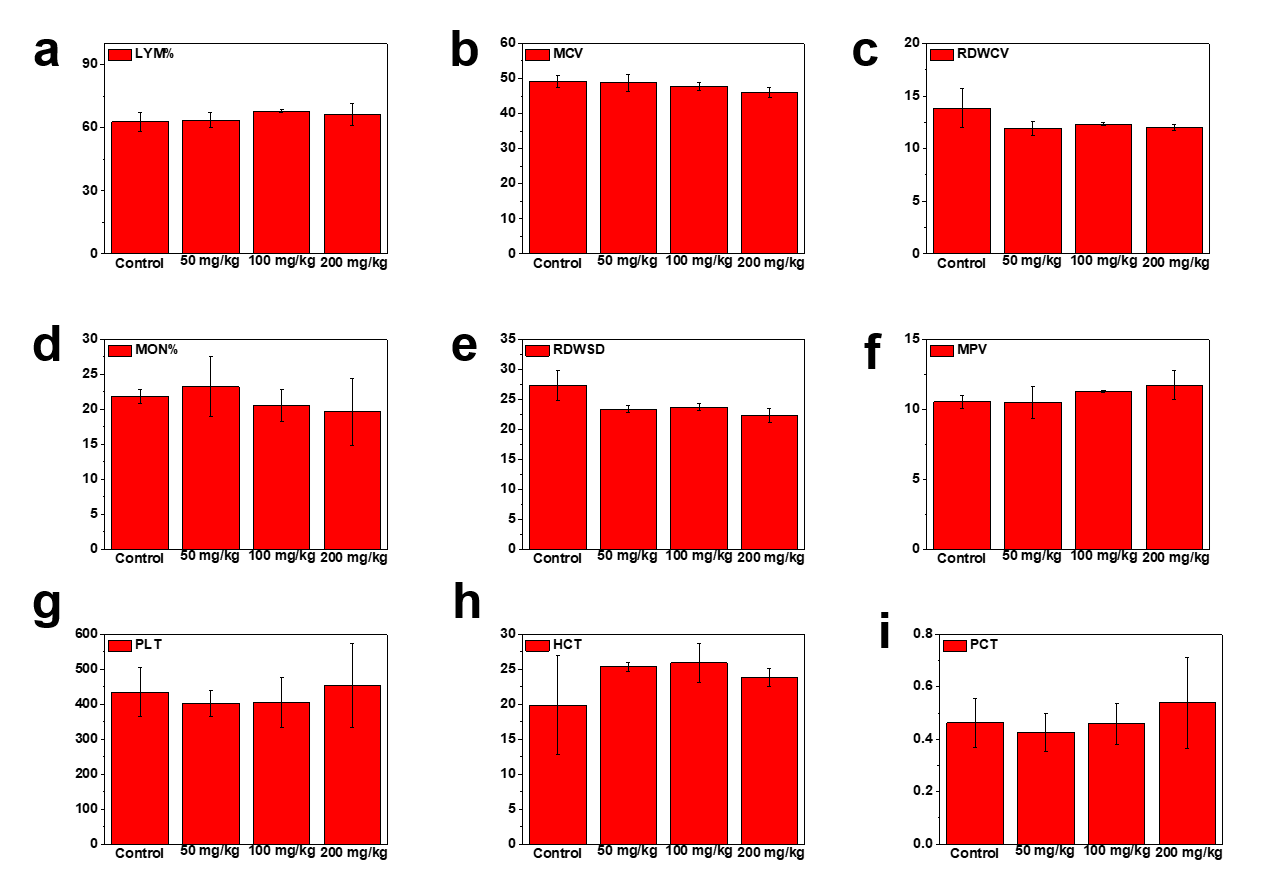


Fig. S12 Blood routine examination including (a) LYM, (b) MCV, (c) RDWCV, (d) MON, (e) RDWSD, (f) MPV, (g) PLT, (h) HCT, and (i) PCT of the mice with the treatment of CMMP (0, 50, 100, and 200 mg/kg) via tail vein injection *in vivo*.


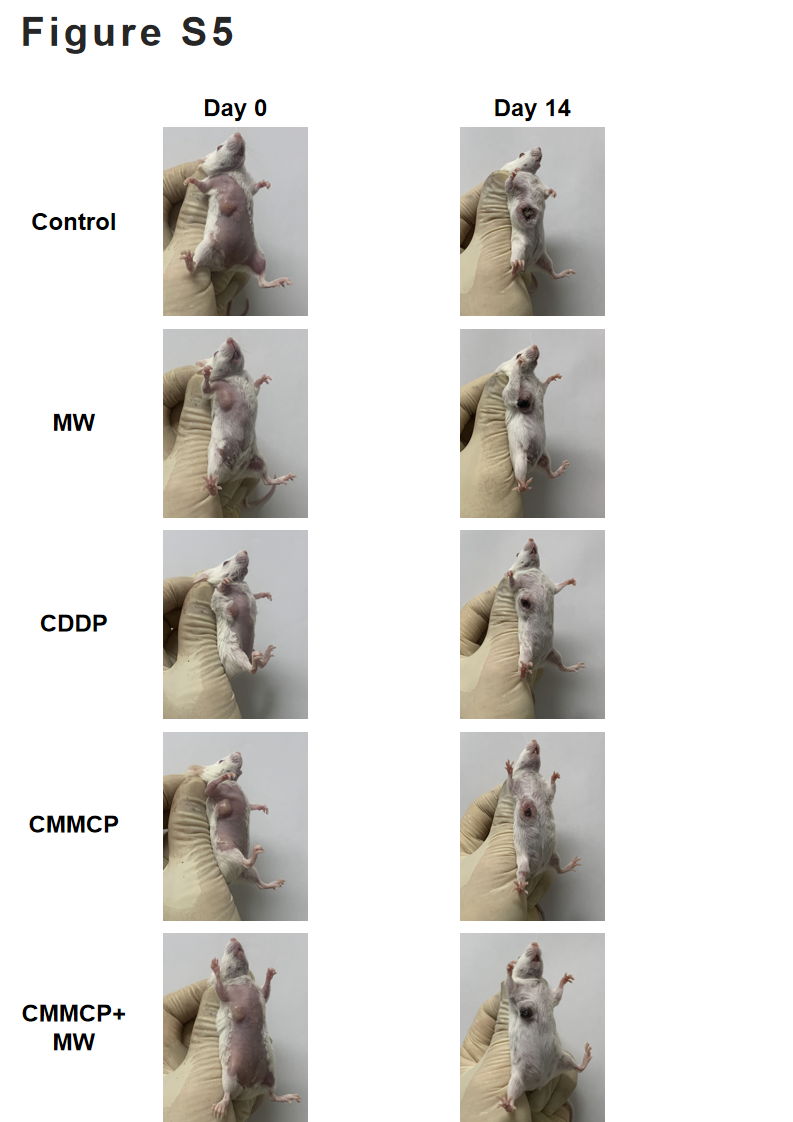


Fig. S13 Photos of the bearing 4T1 tumor mice at 0 and 14 d after MW irradiation.
